# Overlapping signatures of chronic pain in the DNA methylation landscape of prefrontal cortex and peripheral T cells

**DOI:** 10.1038/srep19615

**Published:** 2016-01-28

**Authors:** Renaud Massart, Sergiy Dymov, Magali Millecamps, Matthew Suderman, Stephanie Gregoire, Kevin Koenigs, Sebastian Alvarado, Maral Tajerian, Laura S. Stone, Moshe Szyf

**Affiliations:** 1 Faculty of Medicine, Department of Pharmacology and Therapeutics, McGill University, Montreal, Quebec, Canada; 2 Faculty of Dentistry, Alan Edwards Centre for Research on Pain, McGill University, Montreal, Quebec, Canada

## Abstract

We tested the hypothesis that epigenetic mechanisms in the brain and the immune system are associated with chronic pain. Genome-wide DNA methylation assessed in 9 months post nerve-injury (SNI) and Sham rats, in the prefrontal cortex (PFC) as well as in T cells revealed a vast difference in the DNA methylation landscape in the brain between the groups and a remarkable overlap (72%) between differentially methylated probes in T cells and prefrontal cortex. DNA methylation states in the PFC showed robust correlation with pain score of animals in several genes involved in pain. Finally, only 11 differentially methylated probes in T cells were sufficient to distinguish SNI or Sham individual rats. This study supports the plausibility of DNA methylation involvement in chronic pain and demonstrates the potential feasibility of DNA methylation markers in T cells as noninvasive biomarkers of chronic pain susceptibility.

Chronic pain is one of the most common causes for disability worldwide, with significant global impact on patient quality of life. Despite enormous efforts to find new therapeutic strategies, effective treatments for chronic pain continue to be elusive^1^. There are also no effective ways to predict susceptibility to developing chronic pain in response to injury, which is essential for developing prevention strategies.

Peripheral nerve injury is associated with persistent functional and morphological reorganization of the brain^2^,^3^,^4^,^5^. Among the brain structures implicated in chronic pain conditions, the prefrontal cortex (PFC) is of critical importance in both the affective and sensory components of chronic pain. Changes in this brain area have been reported across many chronic pain conditions as well as in pain-related co-morbidities such as anxiety, depression and cognition^6^,^7^. In rodent models, previous studies by others and ourselves demonstrate the existence of cognitive/emotional deficits many months following nerve-injury^5^,^8^,^9^. However, the mechanisms mediating the long-term effects of injury that result in chronic pain are unknown.

DNA methylation, a covalent modification of the DNA molecule, is involved in stable programming of gene expression during embryogenesis and in mediating the long term effects of experience on genome function and behavioral and physical phenotypes at different time points in life^10^,^11^,^12^,^13^. We therefore hypothesized that changes in DNA methylation are involved in mediating the effects of peripheral nerve injury on chronic pain.

In support of this hypothesis we previously demonstrated that changes in DNA methylation within the periphery can regulate long-term gene transcription in murine models of back pain and humans suffering from chronic back pain^14^. Additionally, we have shown peripheral nerve injury is associated with transcriptome-wide changes in PFC^15^, decreased global DNA methylation in the PFC and amygdala in mice^8^ and can drive the transcription of synaptotagmin within the PFC^16^. Interestingly, environmental enrichment reversed not only nerve injury-induced hypersensitivity but also the global epigenetic reorganization of the rodent brain^17^. However, the genomic landscape of these changes and the particular genes and networks that are involved remains unknown. Identifying targets of DNA methylation changes in chronic pain is critical for establishing the plausibility of our hypothesis as well as for identification of potential candidates for diagnosis and treatment of chronic pain.

A critical question that has implications for further development of therapeutic approaches and diagnostics and predictive markers of chronic pain is whether chronic pain has a systemic manifestation, particularly in the peripheral immune system. Several reports have identified strong links between pain and transcriptional or epigenetic changes in the blood^18^,^19^,^20^. We have previously reported that behavioral experiences that are primarily targeted to the brain, such as maternal care, altered DNA in peripheral T cells^11^,^21^,^22^. We therefore examined here whether DNA methylation changes in T cells are associated with chronic pain and whether these overlap with changes in DNA methylation in the brain.

To address these questions we used a rat model of chronic neuropathic pain induced by peripheral nerve injury (spared nerve injury, SNI) and delineated genome-wide promoter methylation profiles in the prefrontal cortex and in T cells from these animals 9 months post-nerve injury. Our analysis revealed altered DNA methylation levels in thousands of promoters in the PFC between nerve-injured and sham-surgery animals; many of these changes were correlated with the severity of neuropathic pain. Moreover, DNA methylation changes were also associated with neuropathic pain in circulating T cells and strikingly, the majority of the promoters identified as differentially methylated in T cells 9 months post-nerve injury were also affected in the brain. Furthermore, we identified a subset of 11 promoters in T cells that were sufficient to predict rat chronic pain with 80% accuracy and two genes whose methylation levels predicted the intensity of pain-related behavioral changes with a goodness of fit of 0.99. The dramatic changes in the landscape of DNA methylation in the PFC and the functional properties of genes involved support the hypothesis that DNA methylation is a long-term mediator of chronic pain. The striking overlap between the changes in DNA methylation in T cells and PFC supports the feasibility of DNA methylation biomarkers of chronic pain.

## Results

### Peripheral nerve injury-induced changes in DNA methylation in the prefrontal cortex

**Figure 1:**
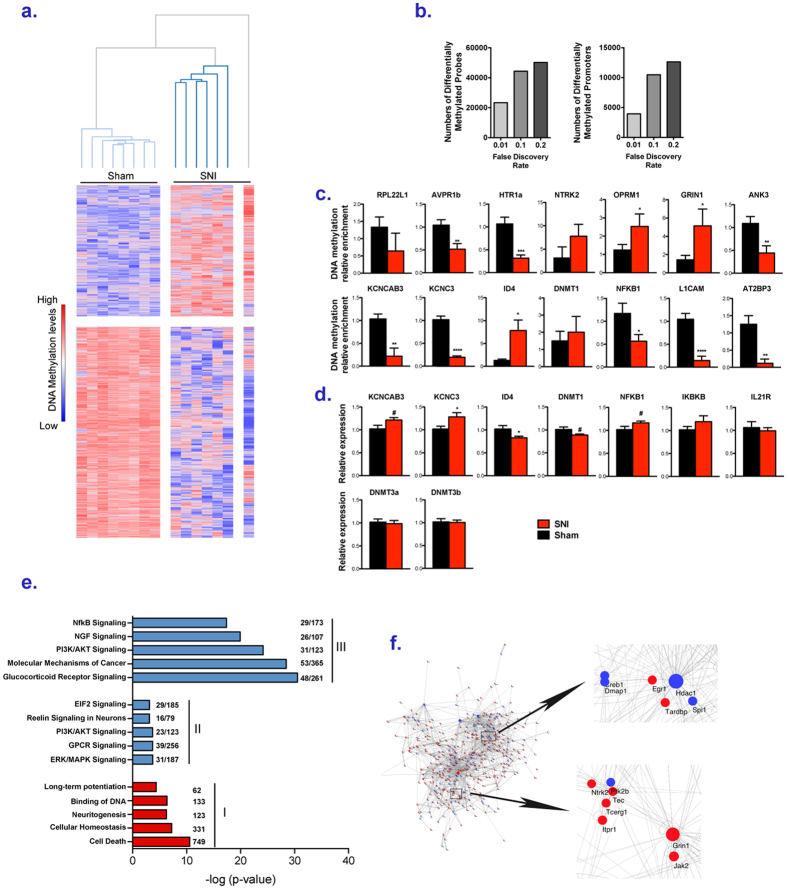
Changes in DNA methylation landscape in PFC in SNI rats. (**a**) Heatmap (row distance metric: Pearson correlation, average linkage) depicting the clustering of the average normalized intensities for each promoter of the microarray probes that were differentially methylated (q < 0.01) between SNI and Sham rats in the PFC. Rows correspond to promoters and columns to animals. Red indicates higher methylation in a row and blue indicates lower methylation. (**b**) Number of probes differentially methylated and of promoters, with at least one probe differentially methylated, at different FDR, between SNI and Sham rats in the PFC. (**c**) QPCR validations of MeDIP-arrays. Each bar plot shows average methylation levels (+/−SEM). Unpaired t test were performed *p < 0.05, ***p < 0.001, ****p < 0.0001. Welch’s correction was applied when variances between groups were significantly different. (**d**) QPCR measurements of relative mRNA expression differences at genes differentially methylated (+/−SEM). Unpaired t test were performed *p < 0.05. Welch’s correction was applied when variances between groups were significantly different. (**e**) Examples of canonical pathways (blue) or biological functions (red) associated with (I) genes differentially methylated (q < 0.01) between SNI and Sham rats in the PFC, (II) genes of metamodule M1 (WGCNA analysis, see supplementary methods) whose methylation levels covary across animals in the brain and that are positively correlated with the SNI condition, (III) genes differentially methylated (q < 0.2) in the brain between sham and SNI rats and with a degree of at least 5 (“hubs”) (NetworkAnalyzer, see supplementary methods). The numbers on the right of the bars indicate either the number of differentially methylated genes associated with a biological function or the number of genes of the metamodule M1 or “hubs” genes associated with a canonical pathway over the number of genes known to be related to the pathway. (**f**) Networks of interconnection between “hubs” obtained using NetworkAnalyzer.

DNA methylation at all annotated promoters in the rat genome and a sample of fully covered genes was determined using methylated DNA immunoprecipitation (MeDIP) followed by hybridization to microarrays and bioinformatics analyses as described in the supplementary methods. Peripheral nerve injury was associated with differential methylation in the PFC of a large number of probes (23,386 out of 400,000) as compared to sham rats (FDR < 0.01), corresponding to 3,946 promoters (defined as −2000/+1000 base pairs from the transcription start site). A heatmap showing unsupervised clustering of all brain samples based on the promoters state of methylation (FDR < 0.01) illustrates the pervasive effect of SNI on the methylation profiles across all the rat brains sampled (Fig. 1a). Among the probes, methylation was decreased in 14,298 probes and increased in 9,088 in SNI animals. Decreasing the level of statistical stringency revealed a staggering number of differentially methylated probes (FDR < 0.1: 44,376 probes associated with 10,483 genes; FDR < 0.2: 50,189 probes associated with 12,643 genes) (Fig. 1b). A number of these differentially methylated regions were validated by qPCR, and the results are consistent with DNA methylation levels estimated by the genome-wide microarrays (Fig. 1c). Analysis of mRNA expression revealed differences in mRNA expression of five genes (KCNAB3, KCNC4, ID4, DNMT1, NFKB1) in opposite directions to the changes in methylation, compatible with a role for these differentially methylated regions in regulating gene expression (Fig. 1d). Four other differentially methylated genes, as assessed by MeDIP-array, did not show differences in expression. This result reflects the complex relationship between DNA methylation and steady-state mRNA levels regulated by various transcription initiation, termination and post-transcriptional processes^23^,^24^.

### Functional organization of the DNA methylation landscape in the prefrontal cortex in response to nerve injury

In order to gain insight into the functional organization of gene networks and pathways whose state of DNA methylation is altered in PFC in chronic neuropathic pain, three different analyses were conducted. First, gene set enrichment analysis was performed, using the Ingenuity Pathway Analysis (IPA) and Genomatix softwares, on the genes whose methylation levels at promoters were the most significantly affected by SNI (FDR < 0.01). IPA revealed significant dysregulation of multiple biological functions including “Cell death” and “Cellular homeostasis”, consistent with elevated cellular stress in the PFC of SNI rats (Fig. 1e, Part I - Table S1). The enrichment in genes related to “Neuritogenesis” and “Long-term potentiation” point to a large impact of peripheral nerve injury on cortical neurotransmission including the dysregulation of dopaminergic, glutamatergic, opioid and serotoninergic systems. Genomatix analysis revealed high enrichments in conditions related to stress and cognitive/emotional deficits such as “Stress” (p = 5.49E-9, 254 genes), “Psychological dependence” (p = 1.61E-7, 11 genes), “Depression adverse event” (p = 6.63E-7, 82 genes), “Anhedonia” (p = 9.03E-6, 15 genes) and “Intellectual disability” (p = 1.32E-5, 100 genes).

**Figure 2:**
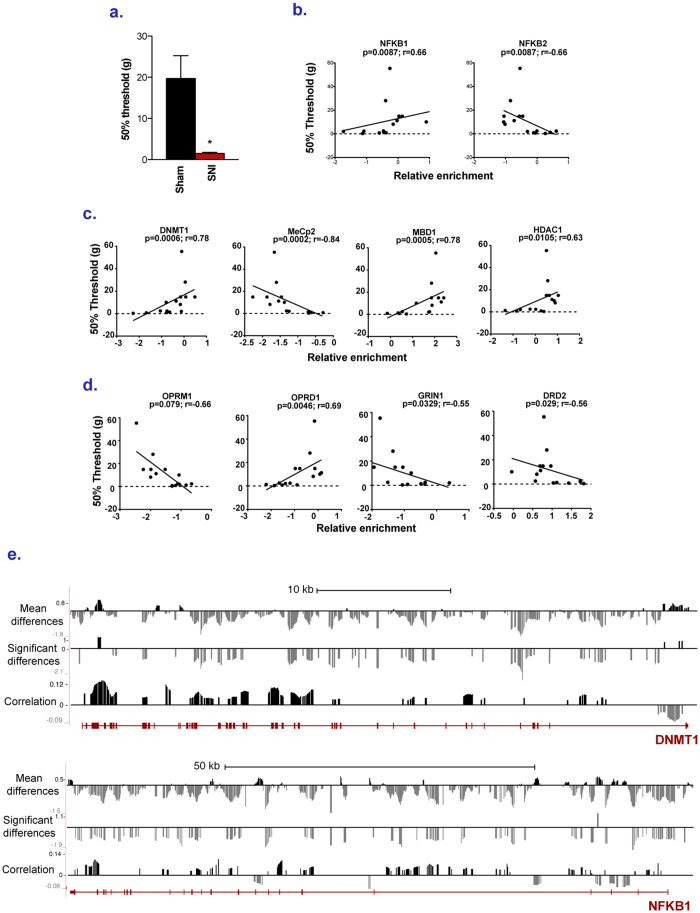
Correlation between DNA methylation states in PFC and mechanical sensitivity. (**a**) Differences in mechanical sensitivity between SNI and Sham rats measured using von Frey filaments. (**b**–**d**) Spearman correlation between mechanical sensitivity of Sham and SNI rats and average DNA methylation levels, measured using microarrays, of differentially methylated probes (q < 0.2) at promoters of (**b**) inflammatory genes, (**c**) epigenetic regulators, (**d**) neurotransmission factors. (**e**) Expanded views from the UCSC genome browser of *dnmt1* and *nfkb1* genes. For each gene, the first track shows average methylation probe fold differences (Log2) between SNI and Sham rats, the second track shows regions significantly differentially methylated (q < 0.2) and the third track show Pearson correlation coefficients between probe methylation levels and mechanical sensitivity of the rats measured using von Frey filaments. The last track shows exons and introns taken from the rat Ensembl RNA reference sequences collection.

In order to uncover relationship structures among differentially methylated genes, weighted gene co-methylation network analysis (WGCNA)^25^,^26^, was applied to differentially methylated gene promoters (FDR < 0.2; 50,189 probes associated with 12,643 genes, see supplementary methods). This analysis identified 82 modules of co-methylated genes across all individual rats whose methylation levels are associated with chronic pain (Fig. 2a, S1) and 81 of them, which comprised 12,526 genes, were significantly associated with nerve injury and with hypersensitivity to mechanical stimuli (Tables S2–3). This result shows that genes are organized in co-methylation modules, which are dependent on the Sham or SNI status. For example, the module arbitrarily named “brown module” was the most significantly associated with the SNI condition (p = 3.07E-10) and the co-methylation profiles of its genes varied in the same range within groups and at a different level between the two groups (Fig. S2). Among these modules, the “brown, orange, blue and dark orange” modules (2319 genes – “meta-module M1”) were positively correlated with the SNI condition and exhibited similar co-methylation profiles, indicating that genes of these four modules could be functionally related and relevant for pain (Fig. S3). Moreover, the meta-module M1 was enriched in genes (821-p = 6.65E-7, Fisher’s exact test) highly associated with the SNI condition; i.e. with a highly significant probe (FDR < 0.01) detected in the promoter. Therefore, a gene set enrichment analysis of the co-methylated genes of the meta-module M1 was performed (Fig. 1e, Part II and Table S4). This analysis revealed enrichments in genes encoding receptors of the glutamatergic, serotoninergic and cannabinoid systems, in genes associated with the ERK/MAPK and PI3K/AKT pathways as well as in EIF2 signaling pathways involved in protein synthesis.

In the third analysis strategy, genes with at least one significantly differentiated probe (FDR < 0.2) were analyzed using the Cytoscape environment^27^ and the NetworkAnalyzer^28^ plugin in order to understand how they interact with other proteins (see supplementary methods). We focused on 2548 genes for which protein-protein interactions data were available in the BioGrid database^29^ and calculated the degree of each in the network, that is the number of interacting partners in the network (Table S5). High degree often indicates that a protein plays a central role in a key cellular function such as signal amplification (kinases)^30^, signal turnover (small GTPases), or gene expression (transcription factors). A total of 409 genes, out of the 2,548 genes mapped on the interactome, have a degree of at least 5 (72% of the genes have a degree ≤ 2). This is consistent with a scale-free organization of cellular networks, in which most of the genes have a few interactions, but a few genes function as “regulatory hubs”^31^. Interestingly, some differentially methylated genes with the highest degrees (e.g. *ubc*, *hdac1*, *grin1*) are part of systems already associated with neuropathic pain^15^,^32^,^33^ supporting an important role for DNA methylation in chronic pain. Moreover, differentially methylated genes with a degree ≥ 5 were highly interconnected (Illustrated in Fig. 1f), indicating they are probably functionally related. Indeed, these genes were found to be enriched in canonical pathways such as PI3K/AKT signaling, the NfkB pathway and in biological functions such as “Cell death” and “Memory” (Fig. 1d, Part III – S4 and Table S4). Finally, 79 genes of the 409 interconnected regulatory “hubs” (degree ≥ 5) were part of the WGCNA meta-module M1 that was most strongly associated with the pain condition. These 79 genes include, for example, the glucocorticoid receptor *nr3c1*, the immune and inflammation regulators *nfkb2*, *ikbkb* and the cAMP response regulator *creb1*.

Taken together, our results show that during the nine months after the induction of neuropathic pain in rats, there was a reorganization of the landscape of DNA methylation in prefrontal cortex, including in cellular pathways and biological functions highly relevant to chronic pain.

### Correlation between pain intensity and gene DNA methylation level in the PFC

We now addressed whether the DNA methylation changes triggered by injury in the PFC are associated with behavioural measures of pain. We therefore determined whether inter-individual differences in DNA methylation in sites that discriminate between the injured and control groups correlate with inter-individual differences in sensitivity to pain across the 16 animals. The persistence of nerve injury-induced hypersensitivity to mechanical stimulation was confirmed using von Frey filaments nine months following SNI surgery (Fig. 2a).

First, the average methylation levels of probes significantly affected at each promoter (FDR < 0.2) were used to calculate correlations between methylation levels of these probes and the mechanical hypersensitivity *in vivo*. Differences in genes involved in NFKB signaling such as *nfkb1* and *nfkb2*, some epigenetic regulators (*dnmt1, mecp2, mbd1, hdac1*) as well as neurotransmission, e.g. opioidergic, glutamatergic and dopaminergic receptors were the most correlated with the pain thresholds (Fig. 2b–d). Table S6 provides additional examples of correlations for a few candidate genes that may be involved in neuropathic pain.

Second, regression analysis revealed that 106,371 probes (FDR < 0.01), corresponding to 7,662 promoters, were significantly correlated with the intensity of mechanical hypersensitivity. Among these significant probes, 22,000 presented a FDR < 10E-4 (Fig. 2e). In addition to promoter regions, the entire loci of several genes were also tiled on the custom microarrays (see Methods). Our DNA methylation analysis showed correlations between changes in DNA methylation and mechanical sensitivity throughout the span of these genes: exons, introns, promoters, intergenic regions and at the 3′ extremities (Fig. 2e). Interestingly, the direction of changes in methylation in promoters is opposite to the changes occurring in the body of the genes, which is consistent with the fact that while methylation in promoters silences gene expression^34^ methylation in body of genes is associated with gene activity^35^.

### Peripheral nerve injury is associated with DNA methylation changes in T cells

**Figure 3:**
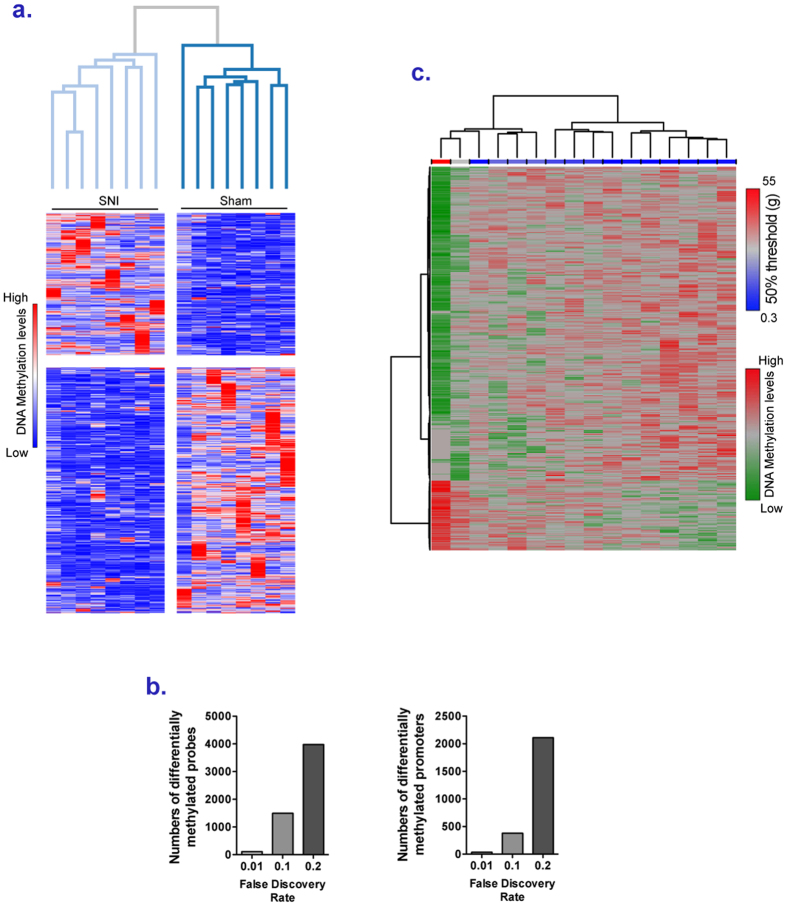
Changes in DNA methylation landscape in T cells in SNI rats. (**a**) Heatmap (row distance metric: Pearson correlation, average linkage) depicting the clustering of the average normalized intensities for each promoter of the microarray probes that were differentially methylated (q < 0.2) between SNI and Sham rats in T cells. Rows correspond to promoters and columns to animals. Red indicates higher methylation in a row and blue indicates lower methylation. (**b**) Number of probes differentially methylated and of promoters, with at least one probe differentially methylated, at different FDR, between SNI and Sham rats in T cells. (**c**) Heatmap (row distance metric: Pearson correlation, average linkage) depicting the clustering of the DNA methylation normalized intensities of the 500 microarray probes that were the most significantly correlated in T cells with the mechanical sensitivity measured using von Frey filaments. Rows correspond to probes and columns to animals. Red indicates higher methylation in a row and green indicates lower methylation. A blue/red horizontal scale indicates the mechanical sensitivity of the rats.

It has been known for decades that DNA methylation patterns are tissue specific^36^, it is therefore common wisdom that DNA methylation associated with chronic pain should be limited to the brain. However, since there is a very well documented inflammatory component to pain^37^, we therefore examined T cell methylation patterns as well (see supplementary methods) and identified 3,982 probes with FDR < 0.2 (2,402 hypomethylated and 1580 hypermethylated in SNI), corresponding to 2,109 promoters (Fig. 3a). At FDR <0.1,1492 probes corresponding to 679 promoters were differentially methylated, and 111 probes corresponding to 34 promoters at FDR < 0.01 (Fig. 3b).

Gene set enrichment analysis on differentially methylated genes (FDR < 0.2) revealed enrichments in pathways including “Cancer” and “Cell death” consistent with cellular stress (Table S7). Moreover, analysis of the T cell array data identified pro- and anti-inflammatory molecules, such as *TNF* (p = 1.49E-5), *IL1β* (p = 2.38E-5) and *TGFB1* (p = 7.22E-4) that have previously been linked to chronic pain^38^,^39^,^40^,^41^. As in the PFC, differential methylation is not only a response to nerve injury but there is a linear correlation between the extent of differential methylation states in T cell and mechanical hypersensitivity (2882 probes at a FDR < 0.01) (Fig. 3c).

### Overlapping DNA methylation changes in prefrontal cortex and T cells

**Figure 4:**
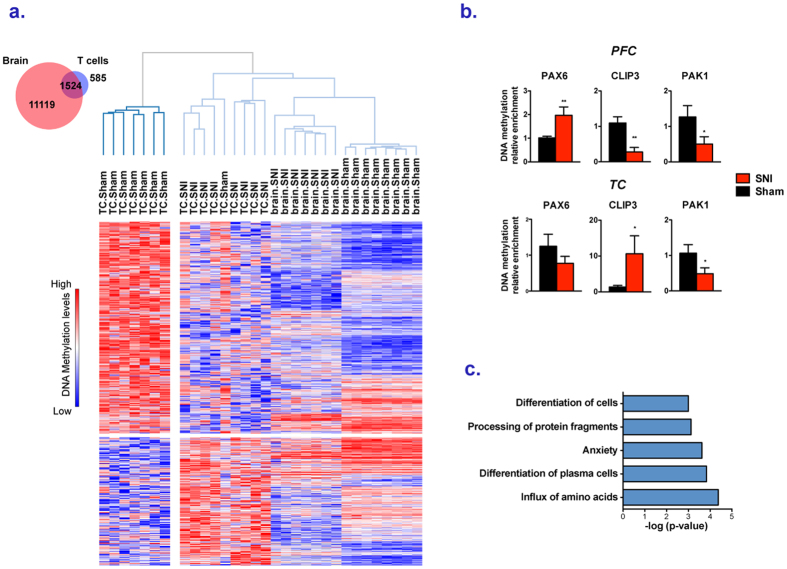
Overlap between differential methylation states in T cells and PFC in SNI rats. (**a**) The Venn diagram shows the overlap between genes with at least one probe differentially methylated (q < 0.2) between SNI and Sham rats in promoters in PFC and T cells. The heatmap (row distance metric: Pearson correlation, average linkage) depicts the clustering of the average normalized intensities for each promoter of the microarray probes that were differentially methylated (q < 0.2) between SNI and Sham rats in T cells and PFC. Rows correspond to promoters and columns to animals. Red indicates higher methylation in a row and blue indicates lower methylation. (**b**) QPCR validations of MeDIP-arrays. Each bar plot shows average methylation levels (+/−SEM). Unpaired t test were performed *p < 0.05, **p < 0.01. Welch’s correction was applied when variances between groups were significantly different. (**c**) Top canonical pathways associated with the genes of the green WGCA module (i.e. genes whose DNA methylation levels covaried across animals in both T cells and the PFC and were positively associated with the SNI condition.

Previous studies have suggested that environmental influences that alter DNA methylation in the brain also impact T cells at similar genomic regions^11^,^22^. We therefore examined the overlap between genes targeted in prefrontal cortex and T cells in response to SNI. We compared the lists of differentially methylated probes (FDR < 0.2) and promoters with at least one significantly differentially methylated probe in the promoter (FDR < 0.2) in SNI vs. sham across the two tissues. A total of 1524 promoters corresponding to 72% of the genes affected in T cells were also differentially methylated in PFC (p = 5.9E-186) (Fig. 4a). 814 identical probes within these promoters (20% of all probes differentially methylated in T cells) were differentially methylated in both tissues (p = 2.15E-13). Clustering analysis of the differentially methylated promoters in both T cells and brain after SNI grouped the samples by tissue as expected^42^ as well as by Sham or SNI status (Fig. 4a).

Consistent with the anticipated tissue specific differences in DNA methylation and the different physiological roles of the immune system and prefrontal cortex in chronic pain, only a portion of the promoters were dysregulated in the same direction in both tissues (43.8% promoters; 44.6% probes). For example, while the differential methylation of *Pak1* was in the same direction in both tissues*, Pax6, Clip3* were differentially methylated in opposite directions, suggesting different roles for the same genes in the systemic response to SNI (Fig. 4b).

Gene set enrichment analysis of the genes affected in both tissues revealed enrichments in pathways including EIF2, calcium and MAPK signaling (P = 1.67E-3, 1.05E-2 and 1.26E-2, respectively) and also in genes with potential upstream regulators such as the pro-inflammatory molecules IL1B (p = 6.06E-4) and TNF (P = 1.7E-3). A WGCNA analysis (see supplementary methods) was applied to the promoters commonly affected in both tissues in order to identify modules of co-methylated genes associated with neuropathic pain in PFC and T cells (Fig. S5a). Among the six co-methylation modules identified in the two tissues, four (corresponding to 447 genes) were significantly associated with SNI and 78% of the genes in these four modules had their levels of methylation affected in the same direction in the brain and T cells by SNI (Fig. S5b). IPA analysis on genes of the green module (Fig. 4c), which is the most significantly associated with SNI revealed enrichments in genes involved in glutamatergic signaling, such as *grin2a, slc1a2*, in inflammatory processes, such as *icam1* and *il21r*, and in calcium signaling, such as *kcnip3* (Table S8). Some of these genes have previously been associated with pain^43^,^44^. This result shows that genes found in co-methylation modules in response to SNI in both tissues are functionally related and are of particular relevance for the neuropathic condition.

### DNA methylation states in T-cells are “predictors” of chronic pain

**Figure 5:**
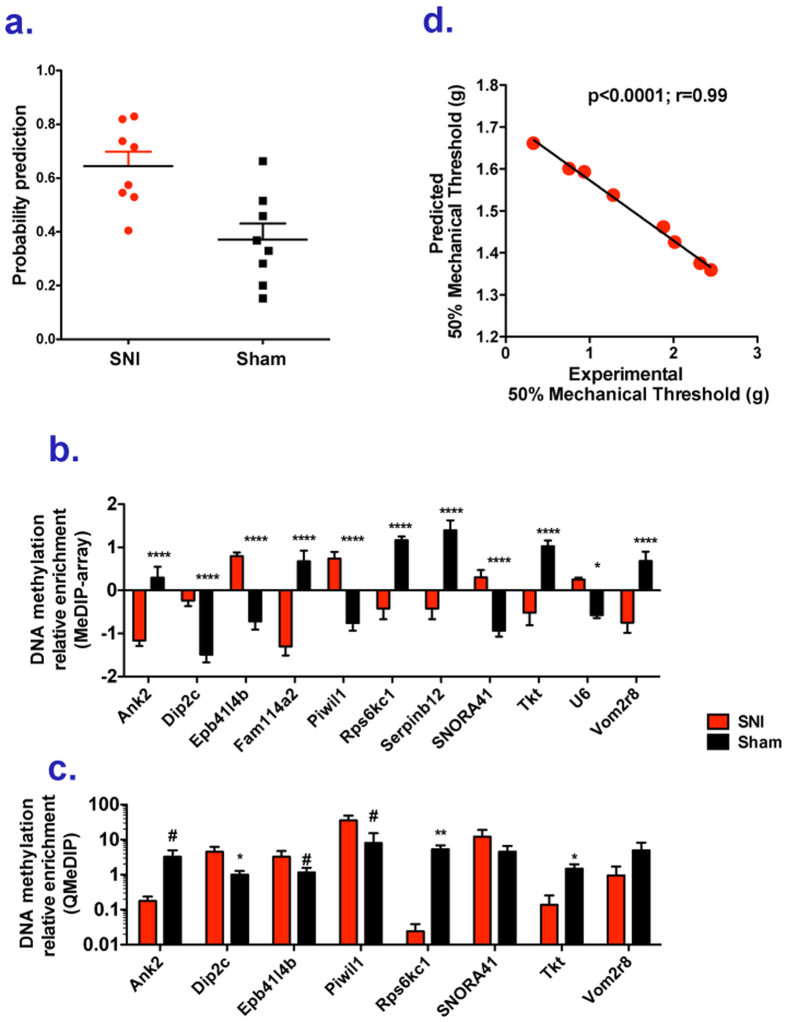
DNA methylation biomarkers predictors of chronic pain in T cells. (**a**) Prediction of rats memberships to SNI or Sham groups based on a likelihood cross-validated penalized logistic regression analysis (see methods section) using averaged methylation levels of differentially methylated probes (q < 0.2) for each promoter in T cells. (**b**) Relative DNA methylation enrichment levels in SNI and Sham rat T cells of differentially methylated probes (q < 0.2) at promoters of genes used for the prediction using the penalized logistic regression (a). Unpaired t tests with Bonferroni correction were performed *p < 0.05, ****p < 0.0001 were performed. (**c**) QPCR validations of MeDIP-arrays. Each bar plot shows average methylation levels (+/−SEM). Unpaired t test were performed ^#^p < 0.1, *p < 0.05. Welch’s correction was applied when variances between groups were significantly different. **(d**) Prediction of rat mechanical sensitivity based on a likelihood cross-validated penalized linear regression analysis using averaged methylation levels of differentially methylated probes (q < 0.2) for each promoter in T cells (y axis) and the mechanical sensitivity determined using von Frey filaments (x axis).

Using penalized logistic regression (see methods section), a prediction model was built based on the list of differentially methylated genes in T cells (FDR < 0.2); this model identified 11 genes that together were sufficient to discriminate SNI from Sham samples. Based on these 11 genes, the prediction for each sample to be a member of the SNI or Sham group is represented in Fig. 5a (0 = Sham; 1 = SNI). Methylation differences for these 11 genes detected by MeDIP-arrays between SNI and Ctrl rats are shown in Fig. 5c. Methylation differences measured by QMeDIP for 8 gene predictors are displayed in Fig. 5c. Not only is the analysis of 11 genes sufficient to distinguish between an SNI or Sham rat, the methylation level of only 2 genes (*srp54a* and x*po4*), were sufficient to predict hypersensitivity to mechanical stimuli in SNI animals. Correlations between these predictions and the actual mechanical thresholds obtained using the von Frey test were nearly perfect (Pearson’s R = 0.99 with p > 0.0001) (Fig. 5d). These data show that DNA methylation markers in T cells could serve as “predictors” of pain sensitivity and potentially chronic pain.

## Discussion

Chronic pain triggered by peripheral nerve injury can persist long after the initial injury. What are the mechanisms that mediate the long lasting effect of nerve damage? In our model of chronic pain rats exhibit mechanical hypersensitivity nine months after spared nerve injury. DNA methylation is a mechanism that can stably alter gene regulation in response to a transient signal^45^. We have previously demonstrated changes in global DNA methylation^8^ and extensive dysregulation of mRNA expression in the prefrontal cortex^15^ in mice with chronic peripheral nerve injury. Alterations in DNA methylation could be just a stochastic footprint downstream of pain-related brain pathology. Our current study strongly argues for the critical involvement of DNA methylation in chronic pain. First, we show that in the PFC, a brain region strongly implicated in chronic pain^6^,^7^, a stunning number of promoters are differentially methylated 9 months after injury (>12,000 individual gene promoters corresponding to more than 50,000 probes, at a FDR > 0.2). These changes are distant both in time and space from the original injury. Second, the changes in DNA methylation are highly organized in functional pathways that have been implicated in pain such as dysregulation of dopaminergic, glutamatergic, opioid and serotoninergic systems and important signaling and inflammatory pathways that include NF kappa B and CREB (Table S1). Third, there is a high coordination of methylation dynamics across many genes across individual animals in several well defined co-methylated modules as determined by a weighted gene co-methylation network analysis (WGCNA) (Fig. S1–2). Fourth, differentially methylated genes encode “regulatory hubs”, which are likely to have a central role in pain such as glucocorticoid and NFkappa B signaling^46^,^47^,^48^,^49^, the ubiquitin–proteasome system^33^ and the dopaminergic, glutamatergic and opioidergic systems^50^,^51^. We show that several of these changes in DNA methylation in promoters are inversely correlated with mRNA levels (Fig. 1d).

A possible criticism of implicating these highly organized changes in the methylome in chronic pain is that they might reflect a general response to injury rather than chronic pain. However, we demonstrate that differential methylation not only differentiates the injured from the non-injured group but that there is a correlation between the extent of DNA methylation changes and quantitative measures of pain severity in thousands of probes in individual animals. Examples of genes correlated with pain severity included major epigenetic regulators *dnmt1* and *dnmt3a*, histone deacetylases *hdac1* and *hdac5*, as well as mu and delta opioid receptors^19^,^52^, ionotropic and metabotropic glutamate receptors and dopamine receptors^50^,^51^. Interestingly, changes in DNA methylation in the mu opioid receptor were previously associated with chronic intake of opioids in humans who suffered from clinical pain^19^. 81 out of 82 modules were affected by the pain condition; the average methylation levels of the genes in each module was either increased or decreased as a function of the severity of the pain (Fig. S2-B). Moreover, coordinated correlation of the extent of methylation change with pain sensitivity levels in individual rats is observed across numerous probes covering entire spans of gene loci as is illustrated for *dnmt1* and *nfkb1* (Fig. 2e).

The correlations between pain sensitivity and changes in DNA methylation in PFC are consistent with clinical studies reporting that both pain duration and intensity are related to changes in PFC structure and function^5^,^53^. To ensure that the impact of chronic pain on the central nervous and immune systems had time to fully develop, rats were evaluated 9 months post-injury, representing a significant proportion of their total lifespan. Additional studies are needed to determine the time course of injury-induced changes in DNA methylation and their relationship to behavior and development of cognitive impairment.

Many of the dysregulated genes and pathways identified have been associated with the negative impact of chronic pain on anxiety, depression and cognition. Gene set analysis revealed enrichments in conditions including depression, anhedonia and long-term potentiation. Furthermore, many of the genes highlighted in Fig. 2 that are correlated with pain sensitivity are also implicated in depression and anhedonia, including the dopamine D2 receptor and the mu- and delta-opioid receptors, and inhibition of HDAC activity in the PFC has anti-depressant actions^54^. Finally, we highlight GRIN1, a subunit of the NMDA receptor, which has a central role in neuroplasticity throughout the CNS. We therefore propose that pain-related changes in DNA methylation are an important mechanism underlying the widespread changes in cortical structure and function observed in many human chronic pain conditions.

Our results implicate wide networks and gene pathways in chronic pain and for the first time show correlation between severity of pain and methylation states of numerous genes in multigenic nodes. While some of the methylation changes may be consequential to other upstream methylation changes, it is highly plausible that many of the changes play a causal role in chronic pain. We therefore hypothesize that the changes in methylation of the genes within networks of interrelated genes will impact the activity of cellular pathways associated with those genes and we speculate that the activity of these cellular pathways may contribute to pain severity. This complex landscape of changes poises challenges for establishing causality as well as for designing therapeutics that could reverse chronic pain. How do we target such a complex network to demonstrate causation and intervention ? One possibility might be designing therapeutic combinations that target entire pathways by acting on “hub” proteins rather than a single or few candidate genes. Our study delineates the pathways and hubs that might be targeted in future experiments. It is interesting to note in this context that examples of ”hub” proteins are the two important DNA methylation enzymes *dnmt1* and *dnmt3a, which* showed gene wide DNA methylation differences that correlate with pain sensitivity. Alterations in activity of these genes might explain the widespread loss in methylation seen in the chronic pain brain: 61% of the probes at promoters (FDR < 0.2) were less methylated in SNI rats. *hdac1* and *hdac5* which are differentially methylated are similarly important in regulating histone acetylation across many genes. These genes might play an upstream role in the hierarchy of epigenetic events involved in chronic pain and might be targets for reversing the “chronic pain” DNA methylation landscape. The changes in DNA methylation in nodal epigenetic regulators strongly implicate epigenetic processes in chronic pain. Our previously work showing that the effects of chronic pain could be reversed by environmental enrichment^17^ support the hypothesis that reversible epigenetic processes such as DNA methylation play a causal role in the chronic pain phenotype and that epigenetic therapy is a potential approach for treating chronic pain.

The utility of DNA methylation biomarkers in neurology and psychiatry is dependent on whether informative changes could be found in peripheral tissues such as saliva or blood. There is a long ongoing discussion on the relevance of changes in DNA methylation in peripheral tissues to phenotypes that are brain-driven because of the inherent tissue specificity of the DNA methylation pattern. One rationale for looking for such markers is the idea that there might be stochastic or experience-driven changes in DNA methylation early in development in common precursors of the neuroendocrine lineage that are maintained through embryogenesis and later in life. Alternatively, it was proposed that the DNA methylation response to experience and the environment, including brain-centered experiences, could target multiple tissues and that the resulting changes in DNA methylation reflect the different physiological roles of particular tissues in the response to the challenge^11^,^22^,^55^.

A major finding of our study is that genome-wide DNA methylation modifications of T cells are also associated with nerve injury, strongly supporting the hypothesis that experience could drive DNA methylation changes in multiple tissues even in adults. This is consistent with the wide range of clinical symptoms associated with peripheral neuropathy including in the immune system *and has broad implications*^37^.

T cell infiltration has been described in the peripheral and central nervous system following nerve injury, the absence of T cells results in reduced hypersensitivity and differential methylation patterns in whole blood samples from humans is associated with altered pain sensitivity^20^,^38^,^56^. The cellular pathways and their potential upstream regulators (e.g. TNF, IL1B, TGFB1) associated with differentially methylated genes in T cells are involved in the maladaptive changes associated with allodynia and hyperalgesia as well as in pain chronicity^38^,^40^,^41^.

Body of evidence demonstrating that life experience results in broad changes in DNA methylation in blood cells is growing rapidly. For example, social economic position in humans^57^, maternal deprivation in rhesus monkeys^11^, maternal prenatal stress^12^,^22^,^58^, maternal depression^21^ and a history of childhood physical aggression are associated with broad DNA methylation signatures in blood^57^ or T-cells^59^ and brain^21^,^22^. The changes observed in the current study may not only contribute to chronic pain but also reflect the unrelenting stress of living with chronic pain. Therefore, the long-term changes in DNA methylation in T cells may play an active role in the pathophysiology of peripheral neuropathies by embedding the transient and chronic exposures to cytokines released after nerve injury in addition to serving as biomarkers of chronic pain.

Most of the promoters (72%) identified as differentially methylated in T cells after nerve injury were also affected in the brain. We identified modules of genes that co-vary in the two tissues. While the methylation profiles in some of these modules were affected in the same direction in the brain and the T cells, others went in opposite direction. This is consistent with the idea that the brain and the immune system play different roles in chronic pain. It is interesting nevertheless that in spite of the different physiological roles, common genes are targeted in both tissues and many genes show similar DNA methylation changes. The changes in DNA methylation in T cells like in the PFC are not just a general consequence of injury since in both tissues the differences in DNA methylation correlate with differences in mechanical hypersensitivity.

Computational prediction models (penalized logistic and linear regressions) run on differentially methylated genes in T cells (FDR < 0.2) identified a set of 11 genes able to differentiate neuropathic animals from controls and a pair of two genes able to predict the mechanical hypersensitivity thresholds in both neuropathic animals and controls. These results suggest that T cell DNA methylation differences might be used in the future as biomarkers not only for the diagnosis and treatment of chronic pain but also for early prediction of high susceptibility to developing chronic pain. It is important to note in this context that discordance in pain sensitivity in identical twins was correlated with DNA methylation differences, including in the TRPA1 promoter, in white blood cells^20^.

In conclusion, these data suggest that persistent pain is associated with broad and highly organized organism-wide changes in DNA methylation, including two critical biological systems: the central nervous and immune systems. These findings reveal potential new avenues for the development of novel therapeutics directed at either the molecular regulation of methylation or at key genes or pathways dysregulated in chronic pain. This work also provides a possible mechanistic explanation for commonly observed comorbidities observed in chronic pain (i.e anxiety, depression). Finally, the sheer magnitude of the impact of chronic pain, particularly in the prefrontal cortex, illustrates the profound impact that living with chronic pain exerts on an individual. Beyond the example of chronic pain, the robust and highly organized DNA methylation changes seen here in response to nerve injury provides some of the strongest evidence to date that experience effects DNA methylation landscapes at large distances in time and space.

## Methods

Detailed methods could be found in supplementary materials and methods online.

### Animals and Spared Nerve Injury (SNI)

Sixteen male Sprague-Dawley rats (Charles River, St-Constant, QC, Canada) weighing 180 g at their arrival were used for all experiments. They were housed 2 per cages, under standard conditions (fresh filtered liter, 12-hour light/dark cycle, temperature: 21 ± 2 °C, humidity: 40–60%). All experiments were performed blind to treatment group. All experiments were approved by the Animal Care Committee at McGill University and conformed to the ethical guidelines of the Canadian Council on Animal Care and the guidelines of the International Association for the Study of Pain Committee for Research and Ethical Issues^60^. Following a one-week habituation period, the spared nerve injury (SNI) model of neuropathic pain (or sham surgery controls) was induced on the left leg (n = 8) under isoflurane anesthesia^61^. Mechanical hypersensitivity was tested nine months after surgery on plantar surface of both hind paws using von Frey filaments as previously described^62^.

### Isolation of PFC and CD3^+^ T Cells and tissue extraction

The rat PFC was dissected following isoflurane anesthesia and decapitation and was defined as Bregma +2.5 to +4.2, +2 mm on either side of the midline and a depth of 5 to 6 mm from the dorsal surface of the cortex. This region, corresponding to the rat mPFC, encompasses the prelimbic and infralimbic subregions and is the closest functional equivalent of the human dorsolateral prefrontal cortex^63^. Brain imaging studies have demonstrated that chronic pain is associated with altered structure, function and connectivity of the human DLPFC^4^,^65^. T cells were isolated from the PBMCs by immunomagnetic isolation using CD3^+^ Dynabeads (Life Technologies, Burlington, ON, Canada) as previously described^11^. CD3 polypeptides play pivotal role in intracellular assembly, surface expression, and signal transduction via the pre-T-cell receptor (pre-TCR) and TCR complexes. CD3 chains are required for thymocyte differentiation and are expressed in virtually all-mature T cells^66^. T-cell and brain DNA and RNA were extracted using the AllPrep DNA/RNA Qiagen kit (Hilden, Germany) and were quantified using the Qubit system (Life Technologies, Burlington, ON, Canada).

### Analysis of genome-wide promoter DNA methylation

The procedure used for MeDIP analysis was adapted from previously published protocols^11^,^22^. A detailed description of the analysis and bioinformatics methods used can be found in supplementary methods. Custom 400K promoter tiling array designs were used for this study (Agilent technologies). Microarray probe sequences were selected to tile at 100 bp spacing all gene promoter regions defined as the genomic interval from −1000 bp upstream to +250 bp downstream of each transcription start site as defined for the rat genome by the Ensembl database (version 60.34b) (http://www.ensembl.org). In addition, several candidate genes were tiled from −50 Kb of the transcription start site to + 50Kb after the transcription end site. These genes included: Arc, Cdk5, Creb1, Crebbp, Ddr1, Dlg4, Dnmt1, Dnmt3a, Drd1a, Drd2, Ehmt2, Fos, Fosb, Gabrd, Gdnf, Gpr156, Gria2, Grin1, Grin2a, Grin2b, Grm2, Grm3, Grm5, Hdac1, Hdac5, Homer1, Homer2, Igf2, LOC367858, LOC691178, Mapk1, Mapk3/4/6/9/10/11/12/13/14/15, Nfkb1, Ntrk2, Q1LZ51, Rac1, Sirt1, Sirt2.

### Gene-specific validation of DNA methylation & mRNA expression analysis

Gene-specific real-time PCR validation of MeDIP-microarray results were performed on the amplified and input bound fractions. Relative enrichment was determined after normalizing from the input fraction in each sample. For QPCR on mRNA, cDNA synthesis was performed using random hexamer primers (Invitrogen) according to the manufacturer’s instructions. Tubuline beta 5 was used as the reference gene. The list of primers is available in Table S9.

### Ingenuity Pathway Analysis (IPA) on differentially methylated genes

For biological function analyses, selected genes were overlaid on the global molecular network developed from information contained in the Ingenuity Pathway knowledge base (www.ingenuity.com) or Genomatix (https://www.genomatix.de). The significance of the association between the datasets and biological functions, canonical pathway or diseases is scored using a p-value calculation (Fisher Exact Test).

### Weighted gene co-methylation network analyze (WGCNA) and module identification

The average methylation level of differentially methylated probes per promoter was first calculated. WGCNA and module identification were performed as described in reference^26^. The modules were then assessed for enrichment in biological functions and canonical pathways using Ingenuity Pathway analysis software.

### Identification of interaction pathways (Cytoscape environment)

Lists of differentially methylated genes (FDR < 0.2) were analyzed using Cytoscape, an open source software^27^, to visualize the interactions between different pathways and the NetworkAnalyzer^28^ plug in installed in Cytoscape to determine degrees for proteins corresponding to the differentially methylated genes^30^.

### Identification of “predictors” of pain severity

Predictors of pain severity were generated and tested using L1-penalized regression^67^ as implemented in the R package ‘penalized’^68^. Methylation input to the regression models were promoter level summaries obtained by taking the average probe intensity across each promoter.

## Additional Information

**Accession codes**: MeDIP and transcription arrays were deposited in GEO accession number GSE70008.

**How to cite this article**: Massart, R. *et al.* Overlapping signatures of chronic pain in the DNA methylation landscape of prefrontal cortex and peripheral T cells. *Sci. Rep.* **6**, 19615; doi: 10.1038/srep19615 (2016).

## Supplementary Material

Supplementary Information

Supplementary Table S3

Supplementary Table S5
