# Supplementary material for: Overlapping signatures of chronic pain in the DNA methylation landscape of prefrontal cortex and peripheral T cells
Source: Sci Rep. 2016 Jan 28;6:19615. doi: 10.1038/srep19615 (PMC4730199; doi:10.1038/srep19615)
Supplement: Supplementary Information [file srep19615-s1.pdf]

Overlapping signatures of chronic pain in the DNA methylation landscape of prefrontal cortex and peripheral T cells

Renaud Massart[1], Sergiy Dymov[1], Magali Millecamps[2], Matthew Suderman[1], Stephanie Gregoire[2], Kevin Koenigs[1], Laura Stone[2]* and Moshe Szyf[1*]

1. Faculty of Medicine, Department of Pharmacology and Therapeutics, McGill University, Montreal, Quebec, Canada
2. Faculty of Dentistry, Alan Edwards Centre for Research on Pain, McGill University, Montreal, Quebec, Canada

Supplementary methods

Animals and Spared Nerve Injury (SNI)

Sixteen male Sprague-Dawley rats (Charles River, St-Constant, QC, Canada) weighing 180g at their arrival were used for all experiments. They were housed 2 per cages, under standard conditions (fresh filtered water, 12-hour light/dark cycle, temperature: 21 ± 2 °C, humidity: 40-60%). All experiments were performed blind to treatment group. All experiments were approved by the Animal Care Committee at McGill University and conformed to the ethical guidelines of the Canadian Council on Animal Care and the guidelines of the International Association for the Study of Pain Committee for Research and Ethical Issues [1]. Following a one-week habituation period, the spared nerve injury (SNI) model of neuropathic pain (or sham surgery control) was induced on the left leg (n=8) under isoflurane anesthesia[2].

Mechanical Hypersensitivity

Following 60 minutes of habituation to the experimental apparatus, mechanical hypersensitivity was assessed on plantar surface of both hind paws using von Frey filaments as previously described (Stoelting Co., Wood Dale, IL). The stimulus intensity ranged from 0.01–60g, corresponding to filament numbers 1.65–5.88. The 50% threshold to withdrawal was calculated using the up-down method as previously described [3].

Behavioral analysis and graphing were performed using GraphPad Prism 6-0. An unpaired t-test was used to compare mechanical hypersensitivity between groups. $P<0.05$ was considered statistically significant.

Isolation of CD3+ T Cells from rat blood

Under deep isoflurane anesthesia, an intracardiac punction was performed for blood collection. Six milliliters was drawn into EDTA-coated tubes and stored at 4°C duringovernight…? .. Peripheral blood mononuclear cells (PBMCs) were isolated through centrifugation with Ficoll-Paque (GE Healthcare, Burnaby, BC, Canada) and washed twice with HBSS (Hanks balanced salt solution, GIBCO)., and T cells were isolated from the PBMCs by immunomagnetic isolation using CD3+ Dynabeads (Life Technologies, Burlington, ON, Canada). The beads were washed 3 times and incubated with the PBMCs for 45 min on a rotator at 4°C. Coated $CD3^+$ cells with the Dynabeads were isolated using a strong magnet (Steam Cell Technology) and washed five times with PBS/FBS. $CD3^+$ cells coated with the Dynabeads were then

frozen at −80°C until DNA extraction. T-cell DNA was extracted with Wizard Genomic DNA Purification kit (Promega) following the protocol of the manufacturer.

Tissue extraction

Animals were sacrificed nine months after nerve injury or sham surgery by decapitation following isoflurane anesthesia. The prefrontal cortex was extracted using the following coordinates (Paxinos and Watson): +1 to +3, -1 to +1, 0 to −2.5. Both hemispheres were pooled for our study since chronic pain that might have an impact on ipsi and contralateral sides of the brain. The samples were then frozen on dry ice and stored at −80 C until use.

DNA and RNA extraction

Genomic DNA and total RNA of brain and T cells were extracted using the AllPrep DNA/RNA Qiagen kit (Hilden, Germany) and quantified using the Qubit system (Life Technologies, Burlington, ON, Canada).

Analysis of genome-wide promoter DNA methylation

The procedure used for DNA methylation immunoprecipitation (MeDIP) analysis was adapted from previously published protocols [4, 5]. Briefly, 2μg of genomic DNA were sonicated using a Bioruptor (Diagenode), and methylated DNA was immunoprecipitated using an anti-5-methyl-cytosine antibody (Eurogentec, Fremont, CA, USA). The DNA-antibody complex was immunoprecipitated with protein G, and the methylated DNA was resuspended in digestion buffer (50 mM TRisHCl pH8; 10 mM EDTA; 0.5% SDS) and treated with proteinase K overnight at 55°C. A fraction of the input DNA obtained after the sonication step and the bound immunoprecipitated fraction were purified, amplified using the Whole Genome Amplification Kit (Sigma-Aldrich, St. Louis, MO, USA), and labeled for microarray hybridization with Cy3-dUTP and Cy5-dUTP, respectively, using the CGH Enzymatic Labeling Kit (Agilent Technologies, Mississauga, ON, Canada) in accordance with the manufacturer ́s instructions. Custom designed tiling arrays were used (Agilent Technologies). All steps of the hybridization and washing were performed in accordance with the Agilent Technologies protocol for chip-on-chip analysis.

MeDIP microarray design, scanning and analysis

Custom 400K promoter tiling array designs were used for this study (Agilent technologies). Microarray probe sequences were selected to tile at 100bp spacing all gene promoter regions defined as the genomic interval from -1000bp upstream to +250bp downstream of each transcription start site as defined for the rat genome by the Ensembl database (version 60.34b) (http://www.ensembl.org). In addition, several candidate genes were tiled from -50Kb of the transcription start site to +50Kb after the transcription end site. These genes included: Arc, Cdk5, Creb1, Crebbp, Ddr1, Dlg4, Dnmt1, Dnmt3a, Drd1a, Drd2, Ehmt2, Fos, Fosb, Gabrd, Gdnf, Gpr156, Gria2, Grin1, Grin2a, Grin2b, Grm2, Grm3, Grm5, Hdac1, Hdac5, Homer1, Homer2, Igf2, LOC367858, LOC691178, Mapk1, Mapk3/4/6/9/10/11/12/13/14/15, Nfkb1, Ntrk2, Q1LZ51, Rac1, Sirt1, Sirt2. All the steps of hybridization, washing, scanning and feature extraction were performed following the Agilent protocols for chip-on-chip analysis (MeDIP). After microarray scanning, probe intensities were extracted from scan images using Agilent's Feature Extraction 10.5 Image Analysis Software. The extracted intensities were then analyzed using the R software environment for statistical computing. Log-ratios of the bound (Cy5) and input (Cy3) microarray channel intensities were computed for each microarray and then microarrays were normalized to one another using quantile-normalization [6] under the assumption that all samples have identical overall methylation levels.

Probe differences between treatment groups or associations with intensity of mechanical hypersensitivity measured in the von Frey test were identified in stages to ensure both statistical significance and biological relevance.

In the first stage, linear models implemented in the 'limma' package [7] of Bioconductor were used to compute a modified t-statistic from the normalized intensities of the probes across all samples between the two groups. An individual probe was called differentially methylated, or associated if the significance of its t-statistic was at most 0.01 (uncorrected for multiple testing) and, for group differences, the associated difference of means between the groups was at least 0.5. For each gene promoter (-1000bp to +250bp of the transcription start site), we calculated the significance of enrichment for high or low probe t-statistics of all probes within the promoter. Significance was determined using the Wilcoxon rank-sum test comparing t-statistics of these probes against those of all the probes on the microarray. The resulting p-values for each gene were then corrected for multiple testing by calculating their false discovery rate (FDR). A gene promoter was then called differentially methylated if its false discovery rate was at most 0.2 and one of its probes was called differentially methylated.

Gene-specific validation of DNA methylation & mRNA expression analysis

Gene-specific real-time PCR validation of MeDIP-microarray results were performed on the amplified and input bound fractions. Relative enrichment was determined after normalizing from the input fraction in each sample. For QPCR on mRNA, cDNA synthesis was performed using random hexamer primers (Invitrogen) according to the manufacturer's instructions. Tubuline alpha 1 was used as the reference gene. SYBR green quantitative PCR (qRT-PCR) was performed using the LightCycler® 480 system (Software 3.5, Roche Molecular Biochemicals). To determine the relative DNA enrichment or concentration of mRNA expression, the $2^{-\Delta\Delta Ct}$ method was used. All data are expressed as group mean ± SEM. The Student's unpaired t-test was used (one-tailed for MeDIP-arrays validations and two-tailed for mRNA QPCR) and the alphalevel was set at 0.05. When variances were significantly different between groups, the Welch's correction was applied. Graphpad 5 software (La Jolla, CA, USA) was used to perform statistical analysis.

Ingenuity Pathway Analysis (IPA) on differentially methylated genes

For biological function analyses, selected genes were overlaid on the global molecular network developed from information contained in the Ingenuity Pathway knowledge base (www.ingenuity.com). The significance of the association between the datasets and biological functions or canonical pathway is scored using a p-value calculation (right-tailed Fisher Exact Test).

Weighted gene co-methylation network analyze (WGCNA) and module identification

WGCNA was first developed to unravel high-order relationships between genes based on their co-expression profiles leading to the identification of modules of biologically related genes and a robust view of the transcriptome organization. WGCNA revealed modules of co-transcribed genes related to physiological and pathophysiological states of the brain [8, 9, 10, 11, 12] or even of specific neuronal populations [13]. This approach was also recently successful at establishing co-methylation modules functionally relevant in multiple human tissues, including blood and brain [14, 15]. WGCNA identifies modules by analyzing the most variable genes of a microarray dataset, determined by their coefficient of variance rather than any sample characteristics such as disease or control status [8]. In our study, the average methylation level of differentially methylated probes per promoter was first calculated. WGCNA then calculated a similarity measure of each pair of genes, called **topological overlap**, indicating the

similarity of their relationships to all other genes. In more details, WGCNA allowed the computation of a matrix containing all pairwise Pearson correlations between the average methylation levels of differentially methylated probes per promoter. The Pearson correlation matrix was transformed into a matrix of connection strengths using a power function, which resulted in a 'weighted' network [16]. **Topological overlaps** are pair-wise measures describing the similarity of two genes' methylation relationships with all other genes in the network. A pair of genes is considered to have high topological overlap if they are both strongly correlated with the same group of genes. Therefore, the modules in our weighted gene co-methylation network are groups of highly correlated genes (i.e. genes with similar patterns of connection strengths or high topological overlap). Modules were identified by applying average linkage hierarchical clustering to the topological overlap measures [16].

Identification of interaction pathways (Cytoscape environment)

Lists of differentially methylated genes (FDR<0.2) were analyzed using Cytoscape, an open source software [17], to visualize the interactions between genes. NetworkAnalyzer [18], a Cytoscape plugin was used to calculated network degree for each gene in the protein-protein interaction network. Degrees refer to the number of interactions involving a protein with other proteins and has been shown to correlate with the essential status of a protein [19].

T cells: Identification of differentially methylated genes to predict the intensity of neuropathic pain

Predictors of pain severity were generated and tested using L1-penalized regression [20] as implemented in the R package 'penalized' [21]. Methylation input to the regression models were promoter level summaries obtained by taking the average probe intensity across each promoter.

Supplementary references

1. Zimmermann M. Ethical guidelines for investigations of experimental pain in conscious animals. *Pain* **16**, 109-110 (1983).

2. Decosterd I, Woolf CJ. Spared nerve injury: an animal model of persistent peripheral neuropathic pain. *Pain* **87**, 149-158 (2000).

3. Chaplan SR, Bach FW, Pogrel JW, Chung JM, Yaksh TL. Quantitative assessment of tactile allodynia in the rat paw. *Journal of neuroscience methods* **53**, 55-63 (1994).

4. Provencal N*, et al.* The signature of maternal rearing in the methylome in rhesus macaque prefrontal cortex and T cells. *J Neurosci* **32**, 15626-15642 (2012).

5. Nieratschker V*, et al.* MORC1 exhibits cross-species differential methylation in association with early life stress as well as genome-wide assciation with MDD. *Transl Psychiatry* **4**, e429 (2014).

6. Bolstad BM, Irizarry RA, Astrand M, Speed TP. A comparison of normalization methods for high density oligonucleotide array data based on variance and bias. *Bioinformatics* **19**, 185-193 (2003).

7. Smyth G. *Limma: linear models for microarray data. In: Bioinformatics and Computational Biology Solutions using R and Bioconductor* (2005).

8. Miller JA, Oldham MC, Geschwind DH. A systems level analysis of transcriptional changes in Alzheimer's disease and normal aging. *The Journal of neuroscience : the official journal of the Society for Neuroscience* **28**, 1410-1420 (2008).

9. Oldham MC*, et al.* Functional organization of the transcriptome in human brain. *Nature neuroscience* **11**, 1271-1282 (2008).

10. Voineagu I*, et al.* Transcriptomic analysis of autistic brain reveals convergent molecular pathology. *Nature* **474**, 380-384 (2011).

11. Cai C*, et al.* Is human blood a good surrogate for brain tissue in transcriptional studies? *BMC genomics* **11**, 589 (2010).

12. Miller JA, Horvath S, Geschwind DH. Divergence of human and mouse brain transcriptome highlights Alzheimer disease pathways. *Proceedings of the National Academy of Sciences of the United States of America* **107**, 12698-12703 (2010).

13. Winden KD*, et al.* The organization of the transcriptional network in specific neuronal classes. *Molecular systems biology* **5**, 291 (2009).

14. Horvath S*, et al.* Aging effects on DNA methylation modules in human brain and blood tissue. *Genome biology* **13**, R97 (2012).

15. van Eijk KR*, et al.* Genetic analysis of DNA methylation and gene expression levels in whole blood of healthy human subjects. *BMC genomics* **13**, 636 (2012).

16. Zhang B, Horvath S. A general framework for weighted gene co-expression network analysis. *Statistical applications in genetics and molecular biology* **4**, Article17 (2005).

17. Shannon P*, et al.* Cytoscape: a software environment for integrated models of biomolecular interaction networks. *Genome research* **13**, 2498-2504 (2003).

18. Assenov Y, Ramirez F, Schelhorn SE, Lengauer T, Albrecht M. Computing topological parameters of biological networks. *Bioinformatics* **24**, 282-284 (2008).

19. Jeong H, Mason SP, Barabasi AL, Oltvai ZN. Lethality and centrality in protein networks. *Nature* **411**, 41-42 (2001).

20. Tibshirani R. Regression shrinkage and selection via the lasso. *J Royal Statist Soc B* **58**, 267-288 (1996).

21. Goeman JJ. L1 penalized estimation in the Cox proportional hazards model. *Biometrical journal Biometrische Zeitschrift* **52**, 70-84 (2010).

Supplementary figures

Figure S1: Clustering by topological overlap (WGCNA analysis, see supplementary methods) reveals modules of differentially methylated genes (q<0.2) between PFC of SNI and Sham rats that are characterized by distinct expression patterns. The *y*-axis corresponds to distance determined by the extent of topological overlap. Dynamic tree cutting was used to identify the modules, generally dividing modules at significant branch points in the dendrogram. Modules with significant overlap were assigned the same color labels.

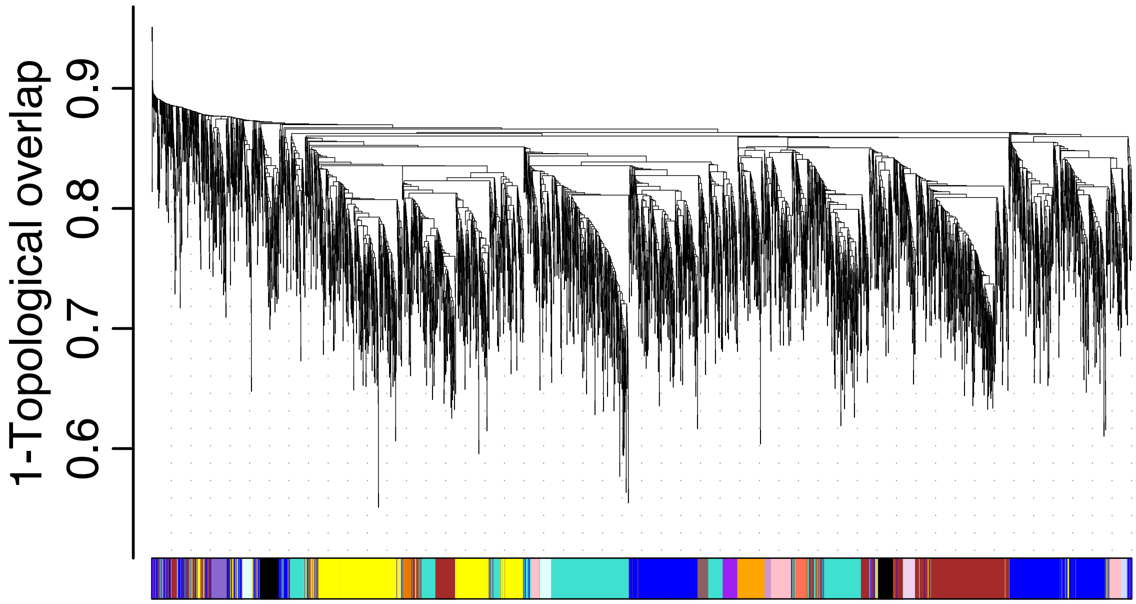

Figure S2: DNA methylation levels of genes of the meta-module M1 that covaried in PFC across animals and whose methylation levels were positively correlated with the treatment (i.e. increased in SNi rats). Each line corresponds to 1 gene.

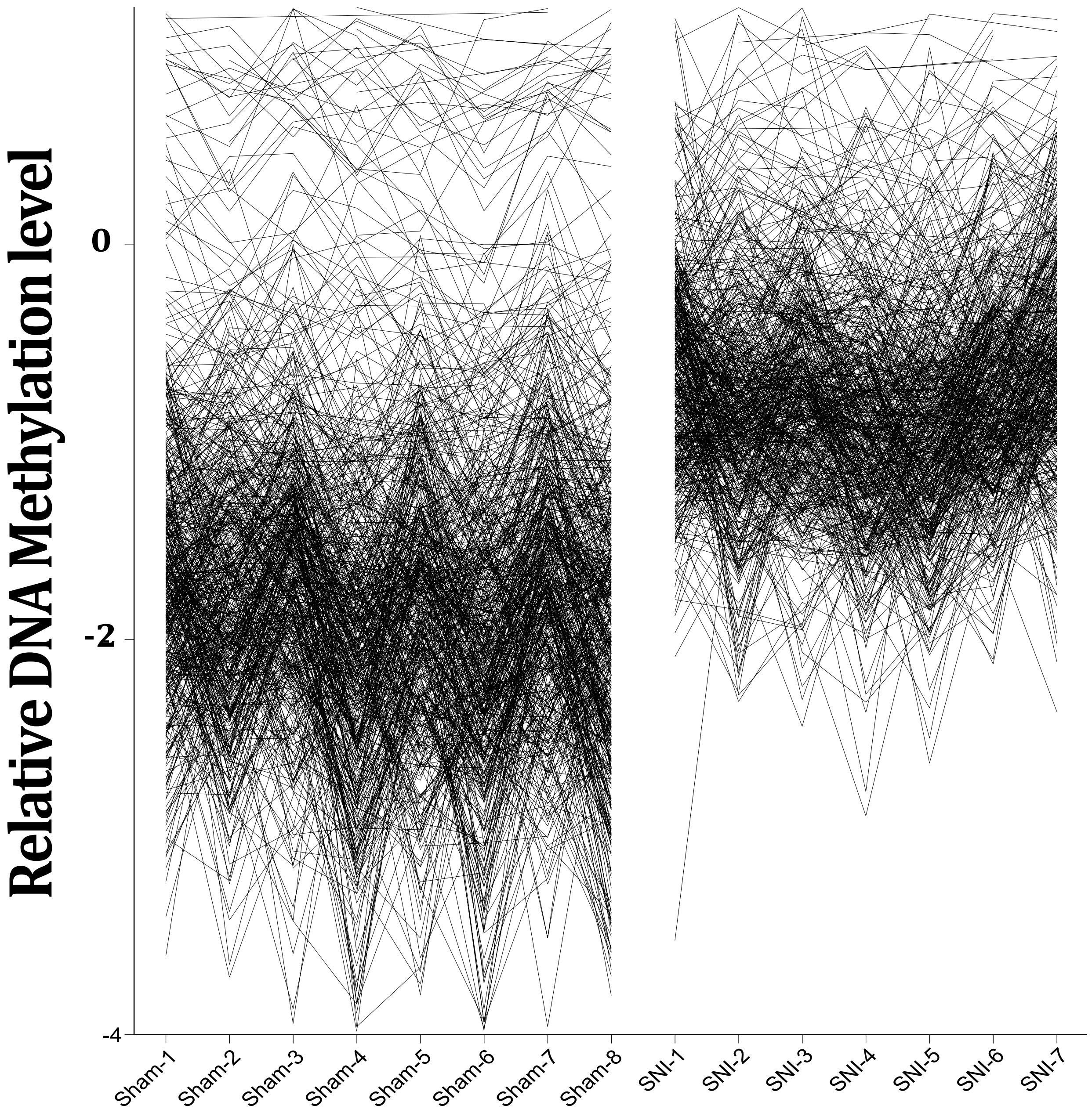

Figure S3: Clustering dendrogram of modules with dissimilarity based on topological overlaps (WGCNA analysis, see supplementary methods) of genes differentially methylated in PFC of SNI rats compared Sham rats. In bold are shown the modules of the meta-module M1, which were the most positively associated with the treatment (i.e. genes whose methylation levels are increased with the SNI treatment).

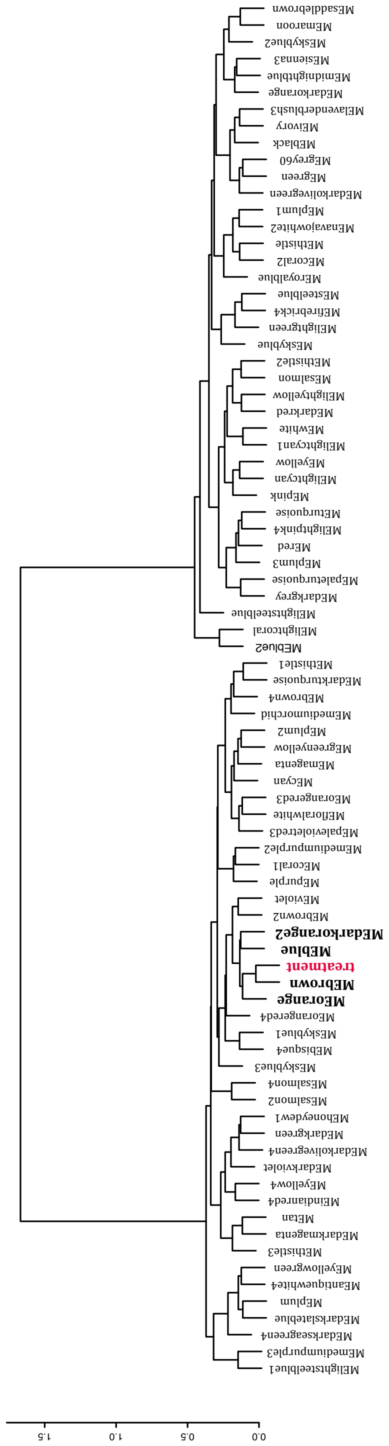

Figure S4: Nfkb signaling based on Ingenuity software. "Hub" genes (with a degree of at least 5) (NetworkAnalyzer, see supplementary methods) that were hyper- or hypo-methylated (q<0.2) in PFC of SNI rats compared to Sham rats are colored in red and blue respectively.

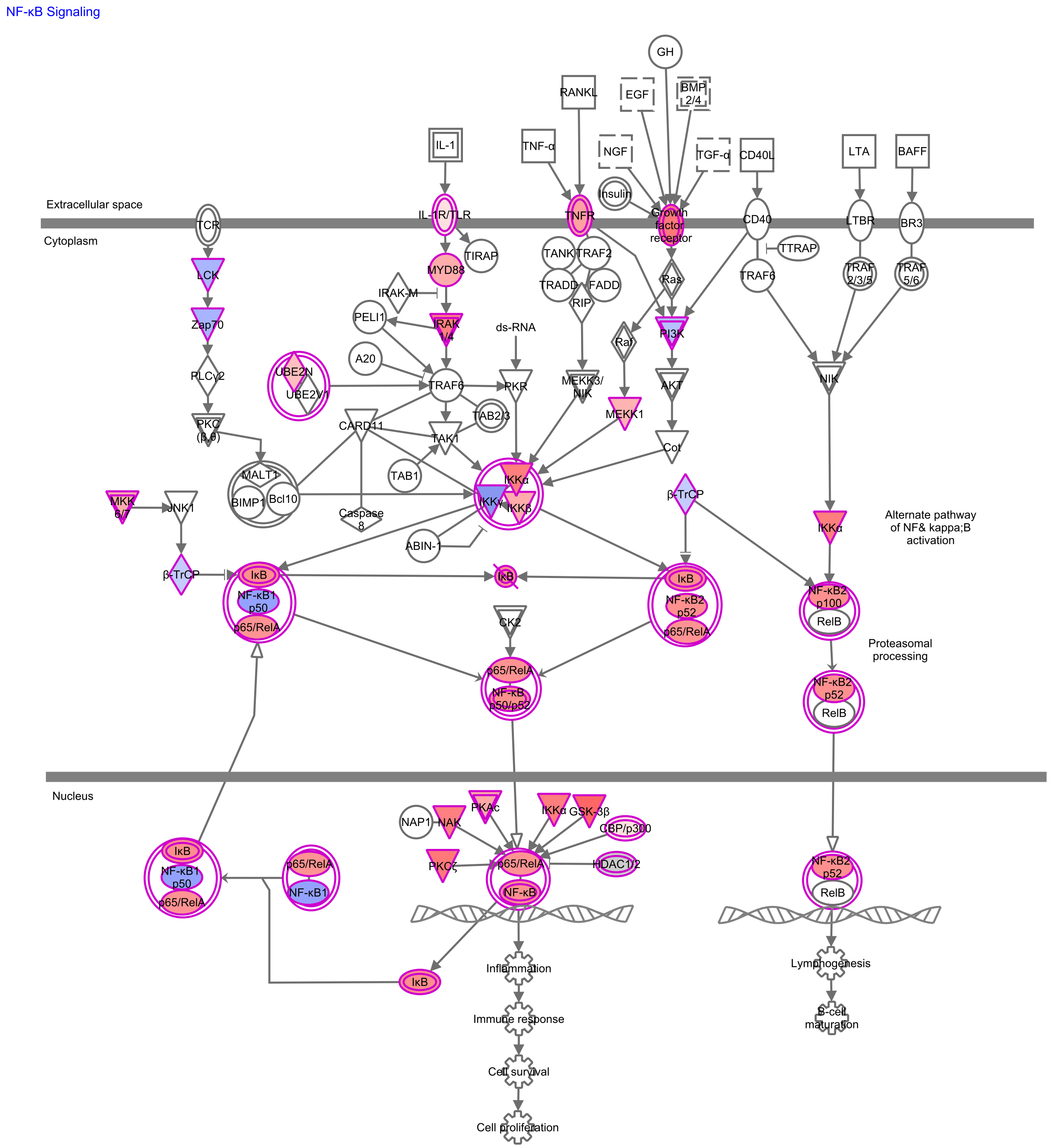

Figure S5: a. Clustering dendrogram of modules with dissimilarity based on topological overlaps (WGCNA analysis, see supplementary methods) of genes differentially methylated in PFC and T cells of SNI rats compared Sham rats. **b.** Associations between modules and SNI condition (treatment) or tissues. Each cell contains the corresponding correlation and p-value. The table is color-coded by correlation according to the color legend.

a.

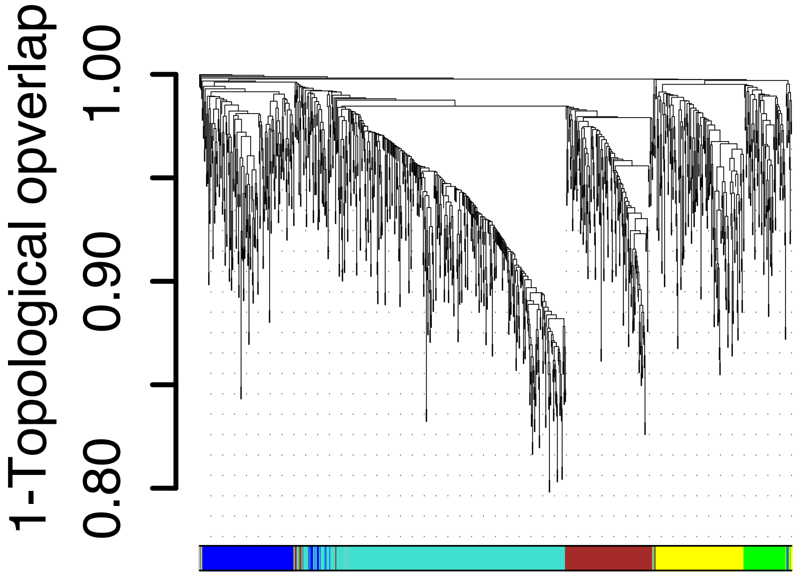

b.

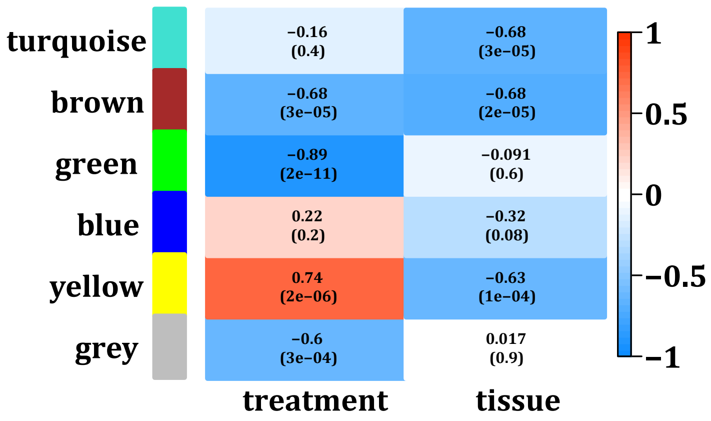

Table S1: Exemples of biological functions enriched in genes differentially methylated (q<0.01) in the cortex

	Categories	p-value	Number of genes	Genes
1	Cell death	2.57E-11	749	AATK,ABCB8,ABCC5,ABCE1,ABL1,ABO,ACTC1,ACVR1B,ACVRL1,ADAM17,ADAMTS1,ADAMTS13,ADAMTS20,ADCY10,ADM,AGRN,AGTPBP1,Agtr1b,AHI1,AIFM1,AIFM3,AKAP12,ALB,ALDOA,ALKBH5,ALOX12,AMPD3,ANK1,ANK3,ANXA1,APAF1,APCS,APEX1,APH1A,AQP11,ARAP3,ARC,ARFIP2,ARHGAP35,ARHGEF6,ARL6IP1,ARNT,ARRB2,ASAH1,ASB2,ATG13,ATG14,ATN1,ATOH1,ATP2B2,ATP2B4,ATP7B,ATR,AURKA,AVP,AXIN1,B2M,BAZ1A,BBS2,BCAS2,BCL2,BCL2L10,BCLAF1,BIN1,BMF,BMP1,BMPR1A,Bmyc,BNIP1,BPI,BRD1,C1QA,C9,CA3,CACNA1A,CACNA1C,CACNB2,CADPS,CALCB,CAMK4,CAMKK1,CANX,CAPN3,CAPNOUS1,CARD9,CASP14,CASP7,CBLB,CCDONC47,CCL4,CCNC,CCT4,CCT8,CD14,CD27,CD300A,CD37,CD3G,CD46,CD6,CD81,CD96,CDA,CDH3,CDK5,CDK8,CDKN2C,CENPI,CFB,CFI,CFL1,CFTR,CHAT,CHD8,CHEK1,CIAPIN1,CIDEB,CIDEC,CIITA,CISD2,CISH,CLASP1,CLCN3,CLCN7,CLEC11A,CLK2,CLU,CLYBL,CMIP,CR1L,CREB1,CREBBP,CRHR1,CSF1R,CSF2,CSK,CSNK2A2,CTBP2,CTGF,CTNNBIP1,CTOUSD,CUL9,CXCL10,CXCL11,Cyb5r3,Cyct,CYR61,DAB2IP,DAPK3,DCLRE1B,DCTN2,DDN,DDR1,DDX25,DDX5,DICER1,DLG4,DLGAP2,DLL4,DLX1,DMC1,DMP1,DNAJA1,DNASE1,DNASE1L3,DNM2,DNMT1,DNMT3A,DNMT3B,DPP7,DRD2,Dst,DUSP4,DVL2,DYNC1H1,DYSF,E2F1,E2F3,ECEL1,EDAR,EFCAB4B,EFNA5,EGLN2,EGLN3,EGR2,EHMT2,EIF3C,EIF3G,EIF4B,EIF4G2,ELANE,ELF4,EMP1,EMP2,EPAS1,ERCC1,ERCC5,ERG,ERN2,ESR1,ESR2,ETV5,F11,FABP1,FAM162A,FANCA,FANCC,FANCF,FANCG,FANCL,FASLG,FASTK,FBLIM1,FBXL2,FBXO17,FBXO5,FCER1A,FCGR2A,FETUB,FGF21,FGF6,FGF7,FGFR1,FGFR4,FLCN,FLI1,FMOD,FNDC1,FOS,FOXO3,FRAT1,FRMD6,FSTL1,G2E3,GABARAP,GABRB3,GABRD,GATAD2A,GCK,GDF3,GDNF,GFI1,GFPT1,GFRA1,GHRH,GIPR,GLIPR1,GLIS3,GLP2R,GLTSCR2,GMEB1,GNAS,GNRH1,GPI,GPM6A,GPR132,GPR65,GPX7,GRB10,GRB2,GREM1,GRIA2,GRIA4,GRID2,GRIN1,GRIN2A,GRIN2B,GRM5,GSDMA,GSK3A,GSTP1,GUCA2B,GZMA,HADHA,HAT1,HCRT,HCST,HDAC1,HDAC5,HDAC9,HES1,HIF1A,HIP1R,HNF4A,HOXB9,HOXC11,HP,HPCA,HPGD,HRAS,HSP90AB1,HSPB11,HSPBAP1,HYAL2,HYPK,IER3,IFNG,IFT57,IFT74,IGF1,IGF2,IGF2R,IKBKAP,IKBKG,IL10,IL13,IL15,IL2,IL21R,IL23A,IL27RA,IL2RG,IL4,INIP,IP6K1,IP6K2,IRF3,Irgm1,ISG20,ITCH,ITGA2,ITGB3BP,ITSN1,JAK3,KCND3,KCNIP1,KCNJ10,KCNJ11,KDELR1,KDM3A,KIFAP3,KLB,KLF10,KLF2,KLF6,KLRB1,Klrk1,KSR1,KSR2,L1CAM,LALBA,LAMA5,LCK,LECT2,let-7,LGALS2,LGALS4,LGALS7,LGALS7B,LGMN,LHB,LIG1,LIMS1,LIN7C,LIPE,LOC100359583,Ptma,LSP1,LTF,LYZ,LZTS1,MAL,MAML1,MAP1LC3B,MAP3K4,MAP4K2,MAPK1,MAPK10,MAPK11,MAPK12,MAPK13,MAPK14,MAPK3,MAPK8,MAPK9,MARK2,MC1R,MC2R,MCF2L,MDC1,MDH1,MECP2,MED21,MED29,MED6,MEF2D,MEFV,MEIG1,MET,MGAT3,mir-10,mir-103,mir-133,mir-135,mir-138,mir-142,mir-143,mir-146,mir-15,mir-154,mir-181,mir-192,mir-194,mir-204,mir-221,mir-29,mir-326,mir-7,mir-8,MITF,Mnt,MPHOSPH9,MPL,MRPS30,MSH2,MSRB2,Mt3,MTA2,Muc4,MUSK,MYBPC3,MYCT1,MZF1,NAPA,NCF2,NCOA3,NCOR2,NCR1,NCR3,NDOR1,NDST1,NDUFAB1,NDUFAF4,NEDD9,NELL1,NEUROD1,NFATC4,NFE2L1,NFIL3,NFKB1,NFKBIB,Ngp,NLRP3,NME3,NME4,NNAT,NOB1,NOL3,NOS3,NPHP4,NPHS1,NPTX2,NR1D1,NR1H4,NR2C1,NR3C2,NSMCE4A,NTF4,NTRK1,NTRK2,NUAK2,NUF2,NUMB,NUPL1,NUPR1,NXF2,NXF2B,OGFOD1,OGT,OLFM1,OPA1,OPRM1,OTX1,PAF1,PAFAH1B2,PAFAH1B3,PAK1,PAK4,PANX1,PARD3,PARG,PARL,PARP14,PAX3,PAX6,PAX8,Paxip1,PCDHGA11,PDCD4,PDE4B,PDE9A,PDIA2,PDPK1,PEX11B,Pgap2,PIGR,PIGT,PIK3AP1,PIN1,PIP,PKD2L2,PKP3,PLAGL1,PLCD3,PLD1,PLD5,PLEC,PLEKHF1,PLG,PLK4,PLRG1,Pmaip1,PNPLA8,POLB,PON3,PPARGC1A,PPAT,PPM1F,PPP1R13B,PPP1R1B,PPP2CA,PQBP1,PRAP1,PRCC,PRDX3,PRDX5,PRKAG1,PRKCB,PRKCG,PRKCQ,PRKCZ,PRKG1,PRKRIR,PRMT1,PROM2,PRPH2,PSEN1,PSMB1,PSMB8,PTGER1,PTGES,PTK6,PTPRA,PTPRE,PTPRF,PTPRH,PTPRO,PTTG1,PUM1,RAB25,RAB28,RAC1,RAI14,RAPGEF2,RASGRP1,RASSF3,RAX,RBBP6,RBM4B,RBM5,RCHY1,REG3A,REL,RHO,RHOG,RND3,RNF17,RNF19A,RNPS1,ROR1,RPL27A,RPRM,RPS6KA3,RPSA,RRAGA,RTN4,RUNX1,RUNX2,RUNX3,RUVBL2,RXFP2,RYR2,S100A1,S100a7a,SAV1,SCARB1,SCGB3A1,SCRIB,SCYL3,SDC1,SDHA,SELL,SELP,SEMA3B,SEMA4D,SENP2,SEPT12,SERPINB4,SERPINC1,SERPINE2,SERPINF2,SFRP4,SGMS1,SGPP1,SHARPIN,SHH,SHISA5,SIAH2,SIGMAR1,SIM2,SIRPA,SIRT2,SKA3,SLAMF6,SLC12A6,SLC19A3,SLC1A2,SLC1A3,SLC20A1,SLC22A2,SLC22A6,SLC28A1,SLC3A2,SLC4A2,SLC6A8,SLC7A9,SLC8A1,SLC8A3,SLIT3,SMAD3,SMPD1,SMYD1,SNCG,SOX17,SOX2,SOX6,SPC25,SPHK1,SPINT2,SPN,SPRY2,SPTLC2,SRCAP,SRPK2,SRR,SRSF1,ST18,STAM,STAT2,STAT4,STAT5B,STIP1,STK17B,STOM,SURF1,SYCP2,SYNE1,SYNGAP1,SYT8,T,TAC1,TAF6,TAP2,TBC1D15,TBCE,TBX5,TBX6,TBXA2R,TCF12,TCF3,TCHP,TCOF1,TCP1,TDRD9,TEK,TFDP1,TFF1,TFPI,TFRC,TGFB1I1,TGFB3,TGM3,THOC2,THRA,THRB,TIA1,TICAM2,TIMM50,TIMP2,TIRAP,TMEM14A,TMEM214,TMEM69,TNFAIP8,TNFRSF13C,TNFRSF17,TNFRSF18,TNFRSF1A,TNFRSF1B,TNKS2,TOP2A,TOPBP1,TOPORS,TP63,TPP2,TRADD,TRAP1,TRIM2,TRIM21,TRIM27,TRIM39,TRPS1,TSC1,TSC2,TSPO,TSSK6,TXN2,TXNIP,TXNRD1,TYMP,TYROBP,UBC,UBE2B,UBE2K,UBE2Q1,UCHL1,UCN,UCN2,UNC13B,UQCRFS1,USP47,VAMP2,VASP,VAV2,VDR,VIP,VIPR1,VPS41,VWF,WAS,WFS1,WNT11,WNT2,XPC,XPO1,XRCC4,YBX2,ZAK,ZBTB18,ZFR,ZNF274,ZNF385A
2	Cellular Homeostasis	5.48E-8	331	ABL1,ACAP1,ACO1,ADAM17,ADAMTS1,ADM,Agtr1b,AIFM3,ALB,ALDOA,ANXA1,APAF1,ARHGAP35,ATG13,ATG14,ATP2B2,ATP2B4,ATP6V0A4,ATP7B,AURKA,AVP,AVPR1B,B2M,BAIAP2,BCL2,BMF,BPI,C1QTNF1,CACNA1A,CACNA1C,CADPS,CALCB,CAMK1,CAPN3,CAPNS1,CARM1,CASP7,CBLB,CCKAR,CCL17,CCL22,CCL4,CCNB2,CD101,CD27,CD300A,CD3D,CD3G,CD46,CD6,CD81,CFTR,CHD4,CHD7,CHEK1,CIITA,CISD2,CISH,CKB,CLCN3,CLEC11A,CLN6,CLTC,CPS1,CR1L,CREB1,CREBBP,CRHR1,CSF2,CSK,CSRP3,CTSD,CXCL10,CXCR5,CYB5R4,CYR61,DAPK3,DBH,DGKZ,DICER1,DLD,DLL4,DMP1,DNMT1,DRD2,DUSP4,E2F1,EGR2,ELF4,ERN2,ESR1,ESR2,FABP1,FANCC,FASLG,FCER1A,FCGR2A,FGF21,FGF6,FGF7,FGFR4,FMR1,FOS,FOXA3,FOXO3,FTMT,FXYD2,FZD8,G6PC3,GCK,GDF3,GDNF,GFI1,GHRH,GIPR,GJA4,GNAS,GNRH1,GPBAR1,GPI,GPR132,GPR15,GPR3,GRB2,GRID2,GRIN1,GRIN2A,GRIN2B,Gsta4,GZMA,HADH,Hamp,HCN1,HCRT,HDAC1,HDAC10,HIF1A,HIVEP2,HNF4A,HNRNPL,HPX,HRAS,HTR2C,IFNG,IGF1,IGF2,IGF2R,IKBKG,IL10,IL13,IL15,IL2,IL21R,IL23A,IL27RA,IL2RG,IL4,INPP5K,IRF3,Irgm1,ITCH,ITGB8,ITSN1,JAK3,KCNJ10,KCNJ11,KCNJ16,KIF5C,KLB,KLF10,KLF2,Klrk1,L1CAM,LCK,let-7,LFNG,LGALS2,LILRB4,LIPE,MAP1LC3B,MAPK1,MAPK13,MAPK14,MAPK3,MAPK8,MAPK9,MARK2,MC2R,MET,MINK1,mir-10,mir-142,mir-15,mir-181,mir-196,MLLT4,MSRB2,Mt3,NCOR1,NCOR2,NCR3,NDUFAB1,NDUFS1,NEUROD1,NFIL3,NFKB1,NKAP,NMUR2,NOL3,NOS3,NR1D1,NR1H4,NR3C2,NTF4,NUPR1,OPA1,OTOP1,OXT,PAK1,PAX6,PCGF2,PDE3B,PDE4B,PDK4,PDPK1,PEMT,PEX5L,PGM1,PKNOX1,PLD1,PLEKHF1,PLG,Pmaip1,PPARGC1A,PPP1R1B,PPP1R3C,PPP2CA,PRDX3,PRKCB,PRKCG,PRKCQ,PRKCZ,PRKG1,PSEN1,PTGER1,PYGL,RAC1,RAD52,RAPGEF2,RASGRP1,REL,RELN,RHCG,RHOT2,RIC3,RPH3AL,RRAGA,RUNX1,RUNX2,RUNX3,RYR2,RYR3,S1PR4,SASH3,SEPT9,SERTAD2,SFRP4,SHH,SIGMAR1,SIRPA,SLAMF6,SLC25A14,SLC34A1,SLC3A2,SLC4A2,SLC8A1,SLC8A3,SMAD3,SMPD1,SORBS1,SOX2,SPHK1,SPN,STAT2,STAT4,STAT5B,STX17,SYPL2,TAC1,TBATA,TBC1D10B,TBXA2R,TCF12,TCF3,TECPR1,TEK,TESC,TFRC,THRA,TICAM2,TLR5,TNFRSF13C,TNFRSF1A,TNFRSF1B,TNKS2,TOP1MT,TRDN,TRIM37,Trpc2,TRPV5,TSC1,TSC2,TXNIP,TYROBP,UCN2,UCP3,VASP,VAV2,VDR,VIP,VIPR1,VPS18,VWF,WAS,Wasl,WFS1,WNK4,XRCC4,ZBTB24,ZBTB7B,Zfp35
3	Neuritogenesis	5.15E-7	123	ABL1,AGRN,AGTPBP1,ANAPC2,ANK3,ARHGAP5,ATCAY,ATP8A2,BAIAP2,BCL2,BRSK1,CACNA1A,CAMK4,CAPNS1,CAPZB,CCKAR,CDK5,CHAT,CLASP2,CLU,CREB1,CRHR1,CRIP1,CSNK2A2,CYR61,CYTH2,DAB1,DAB2IP,DBN1,DCX,DDR1,DICER1,DLG4,DLGAP2,DRD2,Dst,ECEL1,EFNA5,EPS8,ERC2,FGFR1,FMR1,FOS,GALNS,GDNF,GFRA1,GPM6A,GPR126,GRID2,GRIN1,HRAS,IFNG,IGF1,ITSN1,KIAA1598,KIF3B,L1CAM,LIFR,LPPR4,LZTS1,MAGI2,MAPK8,MAPK9,Mbp,MECP2,MET,mir-181,MUSK,NEUROD1,NEUROD6,NFATC4,NFKB1,NLGN3,NTF4,NTNG1,NTRK1,NTRK2,NUMB,OPA1,OPRM1,PACSIN1,PAK1,PARD3,PAX6,PCYT1B,PHGDH,PLD1,PLG,PPP2CA,PREX2,PRKCSH,PRKG1,PRMT1,PSEN1,PTPRE,PTPRF,PTPRK,RAC1,RAPGEF2,RELN,RHOG,ROR1,RTN3,RTN4,RUFY3,RUNX3,SEMA3E,SEMA4D,SHH,SLIT3,STIP1,STMN3,SYNE1,SYNGAP1,TBCE,TNN,TNR,TPBG,UCHL1,UCN,VASP,VIP,WAS
4	Binding of DNA	5.09E-7	133	ABL1,ALB,ALOX12,APEX1,ARNT,AVP,BAZ2A,BCL2,BMPR1A,CARM1,CD101,CFL1,CISH,CLU,CREB1,CREBBP,CSF2,CTGF,CTR9,CYP2A6,DMAP1,DNASE1,E2F1,EGR2,ELANE,ELF4,EPAS1,ERCC5,ESR1,ESR2,FASLG,FGFR4,FLI1,FOS,FOXO3,FYB,FZD8,GFI1,GFPT1,GNAS,GSTP1,GTF2A1,GTF2B,HDAC1,HEY2,HIF1A,HIVEP2,HNF4A,HOXD12,HP,HRAS,IER3,IFNG,IGF1,IKBKG,IL10,IL13,IL15,IL2,IL23A,IL2RG,IL4,IP6K2,IRF3,ITGA2,JAK3,LALBA,LCK,LTF,MAP3K4,MAPK1,MAPK11,MAPK14,MAPK3,MAPK8,MAPK9,MIER1,mir-27,MPL,NCOR1,NFATC4,NFKB1,NFKBIB,NFKBIE,NR0B1,NR1H4,NR2C1,NR2F2,NR3C2,PAK1,PAX3,PBX1,PBXIP1,PIP,PLG,POLB,POU2F1,PPARGC1A,PPP4C,PRKCQ,PSEN1,PTTG1,RAC1,RAD52,RAD54L,RBBP6,REL,RNF17,RPS6KA3,RUNX1,RUNX2,SERPINC1,SMAD3,SMC3,SP140,SPN,STAT4,TAC1,TEF,TFDP1,THRA,THRB,TNFRSF1A,TNFRSF1B,TP63,TRADD,TXNRD1,UBN1,UCN,UCN2,VDR,VIP,XPC
5	Long-term potentiation	4.09E-5	62	ARC,B2M,BAIAP2,CAMK4,CAPNS1,CHRNA2,CLCN3,CREB1,CREBBP,CRHR1,DLG4,DRD2,ESR2,FMR1,GRIA2,GRIA4,GRIN1,GRIN2A,GRIN2B,GRM5,GUCY1A2,HRAS,HTR2C,IFNG,IL10,IL2,IL4,KSR1,MAPK1,MAPK3,NFKBIB,NLGN3,NOS3,NTF4,NTRK2,OPRM1,OXT,PAK1,PCDH8,PCP4,PLG,PPP1R1A,PPP1R1B,PRKACB,PRKCG,PSEN1,PTPRA,RELN,RTN4,RYR3,SERPINE2,SPRED1,SRPK2,SRR,STIP1,SYNGAP1,SYNPO,TNR,TSC2,TSPO,VAV2,WWC1

Table S2: Correlation between topological overlaps of WGCNA modules in the brain (identified using average DNA methylation levels of probes differentially methylated between Sham and SNI rats at q<0.2 for each promoter) and the SNI condition (treatment) or the mechanical sensitivity (Von_Frey).

Modules	pvalue (treatment)	correlation (treatment)	pvalue (Von_Frey)	correlation (Von_Frey)
**MEbrown**	**3.07E-10**	**0.98**	7.99E-03	-0.66
MEthistle2	2.74E-06	-0.91	1.86E-02	0.60
MEmaroon	4.31E-06	-0.90	4.72E-02	0.52
**MEorange**	**1.28E-05**	**0.88**	1.56E-02	-0.61
MEpink	2.04E-05	-0.87	5.89E-02	0.50
MEhoneydew1	2.26E-05	0.87	1.78E-01	-0.37
MEmidnightblue	2.47E-05	-0.87	5.03E-02	0.51
MEdarkred	2.51E-05	-0.87	1.15E-02	0.63
**MEdarkorange2**	**3.10E-05**	**0.87**	5.00E-02	-0.51
MEmagenta	3.29E-05	0.86	1.75E-02	-0.60
MEplum2	3.51E-05	0.86	1.65E-04	-0.82
MEgrey60	3.54E-05	-0.86	8.69E-02	0.46
MEskyblue1	3.76E-05	0.86	2.85E-03	-0.71
MEgreenyellow	3.77E-05	0.86	1.14E-02	-0.63
MEturquoise	4.18E-05	-0.86	4.84E-02	0.52
MEred	4.98E-05	-0.85	4.46E-02	0.52
**MEblue**	**5.04E-05**	**0.85**	1.33E-02	-0.62
MEsaddlebrown	5.55E-05	-0.85	4.31E-02	0.53
MEsalmon	6.30E-05	-0.85	5.31E-02	0.51
MEyellowgreen	6.52E-05	0.85	4.02E-02	-0.53
MEivory	6.76E-05	-0.85	1.77E-02	0.60
MEorangered4	7.34E-05	0.84	1.47E-02	-0.61

MEdarkmagenta	7.78E-05	0.84	5.08E-02	-0.51
MEantiquewhite4	7.83E-05	0.84	1.20E-03	-0.75
MEcoral2	7.88E-05	-0.84	5.43E-02	0.51
MEdarkturquoise	8.36E-05	0.84	4.38E-02	-0.53
MEcoral1	8.77E-05	0.84	4.76E-02	-0.52
MEwhite	9.04E-05	-0.84	6.40E-02	0.49
MEmediumpurple3	9.41E-05	0.84	1.21E-02	-0.63
MEyellow4	9.67E-05	0.84	3.56E-02	-0.55
MEbrown2	1.03E-04	0.84	3.00E-02	-0.56
MEpurple	1.05E-04	0.84	2.35E-02	-0.58
MEsalmon4	1.07E-04	0.84	8.75E-02	-0.46
MEdarkgreen	1.08E-04	0.83	8.41E-02	-0.46
MEplum1	1.16E-04	-0.83	1.81E-01	0.37
MEdarkolivegreen4	1.18E-04	0.83	3.12E-02	-0.56
MElightyellow	1.21E-04	-0.83	9.30E-02	0.45
MEyellow	1.27E-04	-0.83	4.35E-02	0.53
MEfloralwhite	1.29E-04	0.83	3.21E-02	-0.55
MElavenderblush3	1.29E-04	-0.83	2.94E-02	0.56
MEcyan	1.32E-04	0.83	3.71E-02	-0.54
MEdarkorange	1.36E-04	-0.83	6.60E-02	0.49
MEplum3	1.48E-04	-0.83	2.04E-02	0.59
MEblack	1.53E-04	-0.83	2.94E-02	0.56
MEdarkgrey	1.53E-04	-0.83	9.65E-02	0.45
MEdarkolivegreen	1.58E-04	-0.82	6.90E-02	0.48
MEthistle	1.58E-04	-0.82	5.29E-02	0.51
MEgreen	1.61E-04	-0.82	7.69E-02	0.47
MEorangered3	1.67E-04	0.82	3.17E-02	-0.56

MEsienna3	1.72E-04	-0.82	5.08E-02	0.51
MEviolet	1.83E-04	0.82	4.05E-02	-0.53
MEbisque4	1.85E-04	0.82	2.83E-02	-0.56
MElightgreen	2.13E-04	-0.82	8.62E-02	0.46
MEindianred4	2.14E-04	0.82	1.47E-01	-0.39
MEbrown4	2.21E-04	0.81	3.29E-02	-0.55
MEthistle3	2.26E-04	0.81	1.20E-01	-0.42
MEplum	2.34E-04	0.81	5.64E-02	-0.50
MEpaleturquoise	2.34E-04	-0.81	1.65E-01	0.38
MEthistle1	2.97E-04	0.80	3.37E-02	-0.55
MElightcyan1	3.16E-04	-0.80	4.10E-02	0.53
MEroyalblue	3.58E-04	-0.80	5.67E-02	0.50
MEnavajowhite2	3.79E-04	-0.80	8.47E-02	0.46
MElightcyan	3.83E-04	-0.80	4.46E-02	0.52
MEtan	3.87E-04	0.80	1.97E-01	-0.35
MEskyblue3	3.94E-04	0.80	2.68E-06	-0.91
MEfirebrick4	5.27E-04	-0.79	7.83E-02	0.47
MEsalmon2	5.83E-04	0.78	1.40E-01	-0.40
MEsteelblue	6.41E-04	-0.78	1.17E-01	0.42
MElightpink4	8.73E-04	-0.77	3.74E-02	0.54
MEskyblue	1.12E-03	-0.76	2.66E-02	0.57
MEpalevioletred3	1.23E-03	0.75	2.33E-02	-0.58
MEdarkviolet	1.23E-03	0.75	2.19E-01	-0.34
MEskyblue2	1.79E-03	-0.74	4.60E-02	0.52
MEdarkslateblue	1.93E-03	0.73	5.91E-02	-0.50
MElightcoral	2.15E-03	-0.73	2.26E-01	0.33
MElightsteelblue	2.85E-03	-0.71	1.23E-01	0.42

MEmediumpurple2	3.49E-03	0.70	4.43E-02	-0.53
MEmediumorchid	4.17E-03	0.69	1.48E-02	-0.61
MEdarkseagreen4	4.48E-03	0.69	1.38E-01	-0.40
MEblue2	4.50E-03	-0.69	6.31E-02	0.49
MElightsteelblue1	5.32E-03	0.68	9.60E-02	-0.45
MEgrey	9.09E-01	-0.03	6.92E-01	-0.11

Table S4: Examples of canonical pathways or biological functions enriched in differentially methylated genes in the brain that are part of the modules the most positively correlated with SNI (brown, orange, blue, dark orange 2) or that have a degree≥5 (hubs)

1. Meta-module			
**Pathways**	p-value	**Number of genes**	**Genes**
ERK/MAPK Signaling	1.85E-4	31/187	ATF4,CREB1,DUSP2,DUSP4,EIF4E,ELF2,ELF3,ETS1,ITGA2,MRAS,PAK4,PIK3C2G,PIK3R1,PIK3R2,PLA2G2D,PLA2G4B,PPARG,PPP1R14A,PPP2R4,PPP2R1A,PPP2R2C,PPP2R3A,PRKACA,PRKACB,RAPGEF1,RAPGEF3,SOS1,SRC,STAT3,YWHAQ,YWHAZ
G protein coupled receptor Signaling	1.9E-4	39/256	ADRA2B,ADRBK1,Agtr1b,ATF4,CHUK,CNR1,CREB1,DUSP4,FSHR,GNAI3,GRM5,GRM7,HTR2B,HTR2C,IKBKB,MPPE1,MRAS,NFKB2,NPR3,OPRM1,P2RY13,PDE12,PDE6B,PDE8B,PDPK1,PIK3C2G,PIK3R1,PIK3R2,PRKACA,PRKACB,PTGER3,RAP1GAP,RAPGEF3,RGS18,SOS1,SRC,SSTR3,STAT3,SYNGAP1
PI3K/AKT Signaling	2.04E-4	23/123	CHUK,EIF4E,GSK3B,IKBKB,INPP5F,ITGA2,LIMS1,MAP3K5,MDM2,MRAS,NFKB2,PDPK1,PIK3R1,PIK3R2,PPP2R4,PPP2R1A,PPP2R2C,PPP2R3A,RPS6KB2,SOS1,THEM4,YWHAQ,YWHAZ
Reelin Signaling in neurons	7.38E-4	16/79	APBB1,ARHGEF1,CDK5R1,CNR1,GSK3B,ITGA2,LCK,MAP3K9,MAP3K11,MAPK8,MAPK10,NDEL1,PIK3C2G,PIK3R1,PIK3R2,SRC
EIF2 Signaling	7.53E-4	29/185	EIF2A,EIF2S1,EIF3C,EIF4A2,EIF4E,EIF4G2,GSK3B,MRAS,PDPK1,PIK3C2G,PIK3R1,PIK3R2,RPL3,RPL10,RPL22,RPL26,RPL27,RPL28,RPL30,RPL37,RPL39,RPL10A,RPL13A,Rpl39l,RPL7L1,RPS25,RPS26,RPS27A,SOS1
**2. Hubs**			
**Pathways**	**p-value**	**Number of genes**	**Genes**
Glucocorticoid Receptor Signaling	2.80E-31	48/261	ANXA1,BCL2,CEBPB,CHUK,CREB1,CREBBP,ESR1,FOS,GRB2,GTF2A1,GTF2B,GTF2E1,HSP90AA1,HSP90AB1,HSPA2,HSPA9,IKBKB,IKBKG,JAK2,MAP2K1,MAP2K7,MAP3K1,MAPK1,MAPK3,MED1,NCOA3,NFKB1,NFKBIA,NFKBIB,NR3C1,NR3C2,NRIP1,PBX1,PGR,PIK3CB,POLR2A,POLR2B,POU2F1,PRKACA,RAC1,RELA,SHC1,SMAD3,SMARCA2,STAT3,SUMO1,TBP,TSC22D3
Molecular Mechanisms of Cancer	3.58E-29	53/365	ABL1,APC,APH1A,ATR,AURKA,AXIN1,BCL2,BCL2L11,BRCA1,CCNE1,CDK2,CDKN1B,CDKN2A,CREBBP,CRK,CTNND1,DAXX,E2F1,E2F3,FOS,GRB2,GSK3B,HIF1A,JAK2,MAP2K1,MAPK1,MAPK3,MDM2,MYC,NCSTN,NFKB1,NFKB2,NFKBIA,NFKBIB,PAK1,PIK3CB,PRKACA,PRKCZ,PSEN1,PSEN2,PTPN11,RAC1,RELA,SHC1,SIN3A,SMAD3,SMAD5,SMAD9,SRC,SUFU,TCF3,TCF4,XIAP
PI3K/AKT Signaling	6.45E-25	31/123	BCL2,CDKN1B,CHUK,EIF4E,GRB2,GSK3B,HSP90AA1,HSP90AB1,IKBKB,IKBKG,INPP5D,INPPL1,JAK2,MAP2K1,MAPK1,MAPK3,MDM2,NANOG,NFKB1,NFKB2,NFKBIA,NFKBIB,PIK3CB,PPP2CA,PPP2R1A,PRKCZ,RELA,SHC1,TSC1,TSC2,YWHAE
NGF Signaling	1.22E-20	26/107	ATF2,ATF4,CHUK,CREB1,CREBBP,CRK,GRB2,IKBKB,IKBKG,MAP2K1,MAP3K1,MAP3K2,MAP3K4,Map3k7,MAPK1,MAPK3,NFKB1,NFKB2,NTRK1,PIK3CB,PLCG1,PRKCZ,PTPN11,RAC1,RELA,SHC1
NfkB Signaling	4.00E-18	29/173	BTRC,CHUK,CREBBP,GSK3B,HDAC1,HDAC2,IKBKB,IKBKG,IL1R1,IRAK4,LCK,MAP2K7,MAP3K1,MYD88,NFKB1,NFKB2,NFKBIA,NFKBIB,NTRK1,NTRK2,PIK3CB,PRKACA,PRKCZ,RELA,TBK1,TLR4,TNFRSF1A,UBE2N,ZAP70
**Biological Functions**	**p-value**	**Number of genes**	**Genes**
Transcription of RNA	5.19E-112	234	SIAH1,MYC,ESR1,ATF2,MIXL1,Esrra,Foxp1,EHMT2,CREBBP,NFKBIA,CDK2,PRKACA,BACH1,DDX5,PCGF2,ABL1,NR3C1,HDAC1,MAPK1,CISH,BRCA1,PAX3,FOS,TNFRSF1A,TBK1,NRIP1,GPBP1,CCNE1,RUNX2,IRF9,RELA,GRIN1,NR3C2,SIN3A,SMAD5,NR0B2,YY1,DVL3,HTT,TCF12,ATXN3,TAL1,TBP,ETS1,DMAP1,AES,EGR1,PTPN11,JAK2,GTF2E1,ATF4,NKX2-5,SMARCA2,IRF1,NACC1,MAP2K7,SRC,INPPL1,E2F3,TEF,HDAC2,PGR,KAT5,TCERG1,MAP3K1,PIAS3,GTF2B,RAC1,PSEN1,MYD88,DNMT3A,BCL2,USP2,NR1I3,HDAC6,CLOCK,HNF4G,WWP2,APP,SIX1,MED1,MTA2,PARP1,PRKCZ,SS18,MDM4,MYBBP1A,NEUROD1,FOXP3,SP1,CHUK,RUNX1,SUMO1,COPS5,POU2F1,IKBKB,HIF1A,GLI3,SMARCA5,POLR2A,MAP2K,PPAR,SIRT2,HDAC5,SOX2,SERTAD1,HAND2,PRMT5,SETDB1,STAT3,GATAD2A,WHSC1,CEP290,SSBP3,NANOG,CBX7,HCFC1,RUNX1T1,ID2,NDN,SUDS3,PBX2,Tceb2,Cops2,DVL2,DDX20,ESRRB,GSK3B,DAXX,CDKN1B,STUB1,SMAD9,IQGAP1,PTK2B,PBX1,ZAP70,GATA1,NFKB1,SFPQ,TCF4,FOXH1,NFKB2,SMAD3,MEF2C,MSX1,PAX6,IKBKG,APC,HNRNPA2B1,DKK1,MITF,SOCS3,CTCF,TCF3,CALR,PER1,PIN1,GSC,MECP2,PKNOX1,TLE4,NR5A1,NCOA3,PPARGC1A,MAP3K2,CRY2,GRIP1,CDKN2A,PIAS1,PCBD1,THRB,TLE1,SALL1,CEBPB,MED16,CREB1,POLR2B,CCNA2,SPI1,CTBP2,REST,TARDBP,TSC22D3,SFRP1,MYOCD,ZBTB33,TLR4,SUFU,CIITA,PHB2,SHC1,MDM2,GMNN,LCK,MYOG,RBBP7,PCNA,NR1H3,E2F1,MEIS2,CSNK1E,NR0B1,JDP2,PSEN2,TRIM27,CTR9,ASCL1,TFCP2L1,CHD8,WWTR1,SATB2,INPP5D,POLR1A,HAND1,CRK,TCEB1,BTRC,SAP18,LDB1,GTF2A1L,AXIN1,CHD3,ASH2L,OGT,NFKBIZ,NFKBIB,IKZF1,UBE2L3,MAPK3,SP2,KPNA2,CHD4,GTF2A1,SOCS2
Cell Death	2.31E-111	246	POLK,NR3C1,REST,MED1,ATG5,TEC,PPP2R1A,BCL2,APH1A,MYC,MSX1,RAG1,Krt2,GSK3B,PAFAH1B1,PSMD2,KIT,DDX5,FANCL,STAT3,AURKA,CCAR1,NR3C2,RNF19A,Klrk1,ABL1,DAG1,PPARG,HIF1A,CISH,NTRK2,PRKCZ,CDK2,MAPT,SMURF1,CUL5,HSP90AB1,EIF4E,SIX1,DKK1,CLOCK,SP1,BRCA1,CHUK,TAX1BP1,RUNX1,TCF12,IRAK4,SPRY2,PARP1,TBK1,CACYBP,HSPA2,ETS1,HTT,SNCA,SOX2,SHARPIN,PGR,RUNX2,FBXO32,OGT,Ewsr1,MAP1B,JAK2,GRIN1,CRK,LIN7C,SHC1,HAND1,ATR,ESR1,ITPR1,MECP2,SMARCA5,IFT88,NFKBIA,CDKN2A,TCERG1,PCNA,NFKB2,SOCS2,NEUROD1,TOUSC22D3,PIN1,SIAH2,PSEN1,ID2,PPP1CA,NKX2-5,HAND2,RELA,APC,CDKN1B,TRIM27,TCP1,TBP,DNAJA1,E2F3,HOXA9,KRT8,SMN2,PHB2,DAPK3,YWHAE,NDEL1,PAX3,MAPK1,ANXA1,SMARCA2,SFPQ,SUDS3,DDX20,IRF1,Pla2g2a,CHD8,GAPDH,FOS,DAXX,NFKBIZ,STUB1,TARDBP,MAP2K1,SOCS3,KAT5,TNFRSF1A,HSPD1,UBC,EGR1,ATG16L1,CALR,ALS2,CTNND1,MAP3K4,PAX6,SAP18,NEFL,TCEB1,PER1,AHI1,Map3k7,ATF4,PRKACA,JUP,GLI3,ADRBK1,HSPA9,PPP2CA,PIAS3,MDM2,COPS5,MYD88,IKBKB,CDC20,SMAD5,MAP2K7,GMNN,CCNA2,NTRK1,TAL1,NCSTN,TSC2,SRC,RAC1,YY1,POLR2A,MAP3K1,APP,VAMP2,EEF1A1,NCL,ACTB,SFRP1,SUMO1,DNAJC5,ATF2,SOD1,HSP90AA1,UBE2N,NFKBIB,SNAP25,CTBP2,UBE2L3,SALL1,CREBBP,SORBS2,BCL2L11,IL1R1,PSME3,E2F1,TCF4,INPPL1,PRMT1,NR5A1,BTRC,KSR1,GATA1,PXN,PTK2B,SMAD3,NR0B2,PIK3CB,FOXP3,DCC,RUNX1T1,SYVN1,PLCG1,MITF,UBE4B,GRB2,PIAS1,MYH9,LCK,KRT14,RAD18,DNMT3A,HDAC6,PSEN2,PLEC,ZAP70,USP2,PRMT5,INPP5D,ANXA2,SPI1,NPHP4,MEF2C,FBXL2,MAP3K2,PAK1,PDCD6IP,HCST,SIN3A,AES,TYROBP,CBLB,TLR4,NCOA3,THRB,HDAC5,CSF1R,CEBPB,MAPK3,GATAD2A,WWP2,EHMT2,GRIN2A,XIAP,PPARGC1A,SIAH1,NR1H3,KPNA2,NFKB1,DVL2,ATXN3,SLC1A2,BACH1,ATG7,HDAC2,CREB1,CCNE1,SIRT2,CBX7,CTCF,HDAC1,CIITA,NDN,Foxp1,TCF3,NACC1,IKBKG,MDM4,DLG4,ASCL1,TEK,DYNC1H1,CSK,MTA2,WAS,NANOG,RUVBL2,NR1I3,AXIN1,CSNK1E,PTPN11,TSC1
Microtubule dynamics	1.14E-33	106	ESR1,EEF1A1,KIT,CDKN1B,ABL1,MAPK1,SMAD5,DOK1,TSC1,PAX6,NDEL1,APC,NTRK1,PAFAH1B1,SNCA,DPYSL5,CSF1R,SRC,MAP3K1,RAC1,NTRK2,PIN1,MYD88,MECP2,BCL2,HDAC6,NEUROD1,SP1,MAPT,CTNND1,CRK,TLR4,HSP90AA1,DAG1,SMARCA5,PPP2CA,MAP2K1,PPARG,MAP1B,SIRT2,NEFL,NR0B1,DLG1,IFT88,AHI1,MYC,MYH9,DVL2,NFKBIA,GSK3B,IQGAP1,PTK2B,PAK1,BRCA1,TBK1,NFKB1,WAS,DCC,AXIN1,PSEN1,DLG4,APP,CREB1,SS18,CHUK,ENAH,SUMO1,TRIM27,ASAP1,NANOG,WWTR1,MKS1,NPHP4,ASCL1,VANGL2,INPP5D,STAT3,PXN,AURKA,SHC1,SOD1,IL1R1,IKBKB,PRKCZ,SMURF1,UBE4B,DAB1,HDAC2,INPPL1,PTPN11,EGR1,ATG7,RNF19A,PRMT1,HTT,ITPR1,GRIN1,CDC20,TNFRSF1A,FOS,ACTB,ALS2,PRKACA,GAPDH,NDN
Memory	3.55-20	37	ESR1,CREBBP,NR3C1,MAPK1,NFKB1,GRIN1,NR3C2,NFKBIB,PAX6,EGR1,NTRK1,INPPL1,NTRK2,PSEN1,BCL2,HIF1A,MAP2K1,Nedd4,DKK1,APP,CREB1,MAPT,IL1R1,TET1,PSEN2,SNAP25,CSK,PRKCZ,KSR1,MECP2,MAP2K7,SNCA,GRIN2A,ATXN3,HTT,RELA,GSK3B
Ubiquitination of proteins	1.49E-18	36	ATG5,BCL2,RAG1,FANCL,MAPT,SMURF1,CUL5,BRCA1,USP8,HTT,SNCA,NFKBIA,CDKN2A,NDFIP1,SIAH2,DAXX,STUB1,Nedd4,ERCC8,TCEB1,MDM2,MAP3K1,UBE2N,UBE2L3,BTRC,SYVN1,UBE4B,PIAS1,HDAC6,RNF31,WWP2,XIAP,SIAH1,ATG7,AXIN1,LNX1

Table S6: Exemples of genes whose average DNA methylation levels of probes differentially methylated (q<0.2) between SNI and Sham rats at promoters of T cells are correlated with the mechanical sensitivity as measured by the Von Frey test.

Families	Genes	Spearman test
DNA methylation	DNMT1	***
	DNMT3a	**n.s. (p=0.055)**
	MeCP2	***
	MBD1	***
Histones deacetylases (HDACs)	HDAC1	*
	HDAC2	*
	HDAC5	**
	HDAC6	*
Glutamatergic signaling	GRIN1	*
	GRIN2A	**
	SLC5A6	**n.s.**
Dopaminergic receptors	DRD1A	****
	DRD2	*
Opioidergic receptors	OPRM	**
	OPRD1	**
NFKB signaling	NFKB1	**
	NFKB2	**
MAP kinases	MAPK1	*
	MAPK3	**n.s.**
Calcium channels	CACNA1A	**
	CACNA1B	**
	CACNA1C	**

Table S7: Examples of biological functions enriched in differentially methylated genes (q<0.2) in T cells

	Categories	p-value	Number of genes	Genes
1	Cancer	2.28E-09	867	ABCA8,ABCC9,ABCF1,ABCG8,ABHD14B,ABLIM1,ACER2,ACSL4,ACSM2A,ADAD1,ADAMTS13,ADD1,AFF3,AGA,AGR3,AGTR1,AGXT2,AHNAK2,AHSG,AICDA,AIG1,AIM1,AKAP1,AKR1B10,ALAD,ALDH4A1,ALG9,ALOX12B,ALPP,AMPD3,ANK2,ANKRD28,ANKRD46,ANKS1B,ANXA11,ANXA3,ANXA8,ANXA8L1,AP1B1,AP1G1,APBA2,APOA5,ARFGEF2,ARHGAP11A,ARHGAP28,ARHGDIG,ARHGEF12,ARHGEF19,ARHGEF2,ARID1B,ARID3B,ARL5A,ARMC5,ARMC9,ARNT,ARNTL,ARPP21,ASAH2,ASB15,ASCL2,ATAD1,ATF2,ATL3,ATP10D,ATP2A3,ATP2B2,ATP2B3,ATP2B4,ATP6AP1,ATP6V0D2,ATP8B3,AVP,B3GNT3,B4GALT1,B9D1,BEND5,BEND7,BFSP1,BICD2,BIN2,BLZF1,BMP1,BMP3,BMPER,BNIPL,BRCA2,BRF1,BRPF3,BSG,BTBD16,BTRC,C12orf60,C15orf40,C16orf62,C16orf92,C17orf49,C19orf47,C1orf64,C1QTNF7,C20orf27,C2CD2L,C2orf16,C2orf42,C3orf30,C6orf118,C6orf89,C7orf60,CA6,CABP2,CABP4,CACNA1B,CAD,CALCB,CAMTA1,CAND1,CANT1,CAP1,CAPS2,CAPZA1,CASP3,CASQ2,CATOUSPER1,CBX7,CCDONC88C,CCDONC90B,CCKAR,CD226,CD2BP2,CD40,CD47,CD80,CD96,CDONC14A,CDONC20,CDC25B,CDC42EP1,CDC42EP4,CDH23,CDH24,CDK5RAP2,CDKN2B,CDX1,CDYL,CEP57,CEP72,CFLAR,CHST15,CHST8,CIT,CKS2,CLCA1,CLCN5,CLEC3A,CLEC3B,CLNS1A,CLPS,CNOT3,CNRIP1,COL12A1,COL2A1,COL3A1,COPS5,COPZ1,CORO2B,COX6A2,CPB2,CPSF2,CPT1C,CREBBP,CROT,CRTAC1,CSNK2A1,CST3,CTBP2,CTGF,CTIF,CTNNB1,CTSC,DAGLB,DAPK1,DAXX,DCBLD1,DCC,DCK,DCLK3,DCTN5,DDX10,DDX54,DEK,DENND2C,DEPDC1B,DERL2,DGKH,DGKK,DHRS3,DHX57,DIP2C,DIS3,DKK4,DLG2,DMBT1,DMPK,DMRT1,DNAJB1,DNMT3A,DNMT3B,DNMT3L,DOCK2,DOCK3,DOCK6,DOK4,DOLPP1,DONSON,DPEP1,DPY19L4,DPYS,DQX1,DSE,DSPP,DYNLRB1,DYSF,EDNRA,EEF1D,EFTUD2,EIF3A,EIF3K,EIF4E,ELK1,ELMOD1,EMD,ENC1,ENO4,ENPP3,ENPP4,ENTHD1,ENTPD6,EPB41L4B,EPHB1,EPHB6,ERBB2,ERLIN2,ESPL1,ESPN,ESPNL,ESR1,ETV5,EVX2,EXOC2,EXOSC4,F13A1,FABP1,FAIM2,FAM103A1,FAM110D,FAM120C,FAM131B,FAM193A,FAM19A4,FAM73B,FAM83F,FAM92B,FANCG,FASTKD1,FAT1,FBXL3,FBXO11,FBXO32,FBXW2,FCER1A,FCGBP,FECH,FEN1,FGFR2,FGFR4,FHL3,FIGNL1,FIS1,FKBP5,FNDC8,FOXO3,FOXO4,FRAS1,FSD1L,FSIP2,FSTL5,FUK,FYTTD1,GAL3ST3,GALC,GALNTL6,GAN,GAS6,GATA5,GEMIN4,GHRH,GHRHR,GJB5,GLI3,GLTSCR2,GLYAT,GNAL,GNAS,GNB2L1,GNRHR,GPBAR1,GPR123,GPR126,GPR135,GPR158,GPR22,GPR34,GPR55,GPR75,GPRC5B,GPRIN2,GRAMD1A,GREB1,GRIA2,GRIN2A,GRK1,GRM1,GSN,GSTA1,GSX2,GTF2A1,GUCY2F,GUK1,HAND1,HDAC9,HEATR1,HECTD1,HECTD4,HES5,HIC2,HIP1,HIVEP2,HIVEP3,HK3,HLTF,HMX2,HNF4A,HNRNPA1,HPD,HPGDS,HRH2,HS6ST2,HSPA5,HTR2B,ICAM1,ID4,IER2,IGFBP2,IGSF11,IGSF9,IL18R1,IL1R1,IL1R2,IL21R,IL24,IL36RN,IL6R,IL6ST,IL7,ILDR1,ILDR2,IMMT,ING3,INPP5B,INPP5F,INSIG1,INSR,IPO4,IQCJ,SCHIP1,IREB2,IRGQ,ITPRIPL1,JAZF1,KCNA1,KCNA5,KCND3,KCNJ9,KCNQ2,KCNV1,KCP,KEAP1,KIAA0226L,KIAA0232,KIAA1549,KIAA1804,KIAA2026,KIF2B,KIR3DL3,KIRREL,KLB,KLHL14,KLK1,KPNA4,KRT23,KRT85,KRTAP4-11,LAIR1,LAMA5,LCAT,LCP1,LGALS7,LGALS7B,LIG1,LILRB4,LNX1,LPAR1,LPAR3,LPAR4,LPCAT1,LPO,LPPR2,LRFN3,LRP8,LRRC16B,LRRC26,LRRC66,LRRTM2,LRSAM1,LUZP1,LY86,LYN,LYSMD1,MADD,MAFK,MAG,MAML3,MAN1A1,MAN2A2,MAP3K1,MAP4K3,MAPK1,MAPK10,MAPK4,MAPKBP1,MARS2,MBTPS1,MBTPS2,MCEMP1,MCMBP,MED23,MEIS2,MICAL2,MICU3,MINK1,mir-1,mir-10,mir-130,mir-17,mir-181,mir-196,mir-204,mir-22,mir-224,mir-28,mir-29,mir-296,mir-30,mir-329,mir-34,mir-370,mir-7,MLH1,MLLT4,MMP1,MMP21,MMP8,MOCS2,MPV17,MRE11A,MRPL47,MRS2,MSH3,MSMB,Mt1,MTF1,MTIF2,MTMR4,MUC5B,MUM1,MUM1L1,MYBL2,MYH8,MYL9,MYNN,MYO1D,MYO1F,MYO6,MZF1,NAPEPLD,NAV2,NAV3,NBN,NCL,NCOA4,NDRG4,NDUFV1,NDUFV2,NETO1,NEU3,NEU4,NEUROG1,NEXN,NFU1,NMD3,NMNAT2,NOL10,NOTCH4,NOVA1,NPHP4,NPY,NR5A2,NR6A1,NRD1,NRG2,NRIP3,NTRK3,NUP93,NUPL2,NXF2,NXF2B,OAZ1,ODF3,OLIG2,OPN4,OR10J3,OR2L2,OR51A7,OR51F1,OR52B6,OR5A1,OR5W2,OR6A2,OR8A1,OR8D4,OTOL1,OTUD6A,OTUD7A,P4HB,PACSIN2,PAK1,PALB2,PANX2,PAPSS2,PAQR4,PARP16,PBRM1,PCCA,PCDH11X,PCDH8,PCDHGA2,PCNXL3,PDCD6,PDDC1,PDE10A,PDE2A,PDE4C,PDE4DIP,PDP2,PDSS2,PDZK1IP1,PEX11A,PF4,PGAP1,PGC,PHACTR2,PHF1,PHF21B,PHF3,PIK3C2A,PIK3C3,PINX1,PIP5K1A,PIWIL1,PLA1A,PLA2G15,PLA2G4E,PLA2G4F,PLEC,PLEK,PLEKHA1,PLEKHM3,PLK1,PLSCR1,PMEPA1,PMF1,BGLAP,PMP22,PNKD,PNPLA4,PNRC1,PODNL1,PODXL2,POMT1,POU3F2,PPAP2B,PPARG,PPCDC,PPM1A,PPM1H,PPP1R11,PPP1R16A,PPP1R7,PPP2R1A,PPWD1,PRAM1,PRCC,PRDM10,PRKAB1,PRKAG3,PRKCG,PRMT3,PRPSAP2,PRR14,PSME3,PSRC1,PTF1A,PTGS1,PTPDC1,PTPN22,PTPRN,PYCARD,RAB3IL1,RAB40B,RAD18,RAD51,RAD51C,RALBP1,RB1,RBBP8,RBMS3,RCAN2,RDH5,RECK,REP15,REPS2,REXO1,RFX5,RGMA,RGS2,RHBDD2,RHBDF2,RHOQ,RIBC1,RLTPR,RND2,RNF112,RNF146,RNF182,RNF183,RNF44,RNF5,RNF6,RPGR,RPL11,RPL28,RPLP1,RPS11,RPS18,RPS6KA2,RPS6KC1,RRM2,RSPH3,RTEL1,RTN4,RUNX1T1,RUVBL1,RXFP2,RYR2,S1PR5,SARS2,SBSN,SCAF4,SCG2,SCN10A,SCNN1A,SCRN2,SCYL3,SDCBP2,SEBOX,SEC23IP,SEC24B,SEMA4B,SEMA6C,SERPINB12,SERPINB6,SERPINC1,SERTAD2,SET,SETD4,SETX,SGCG,SH3D19,SHISA7,SHQ1,SHROOM1,SIGLEC10,SIGMAR1,SIM1,SIRT7,SKIL,SLAMF6,SLC12A5,SLC16A12,SLC17A8,SLC1A4,SLC20A1,SLC22A18,SLC25A11,SLC25A21,SLC25A37,SLC25A38,SLC27A3,SLC29A4,SLC2A1,SLC2A9,SLC39A12,SLC6A11,SLC6A18,SLC6A6,SLC7A1,SLC7A14,SLC7A4,SLITRK1,SMARCA4,SMNDC1,SMTNL2,SMYD4,SNCA,SNRK,SNTB1,SNTN,SOX17,SPAG16,SPAG5,SPHK1,SPHK2,SPHKAP,SPIB,SPN,SPP2,SPRR3,SPTAN1,SPZ1,SRP54,ST18,STEAP4,STK11IP,STK35,STK39,STMN3,STOM,STOML2,SULT2B1,SUV420H1,SYK,SYNE2,SYNRG,SYT16,TAAR5,TAF1D,TBC1D10B,TBC1D2B,TBXAS1,TCEA2,Tcf7,TCIRG1,TDGF1,TECPR1,TEKT4,TFDP1,TFDP2,TFG,TGM2,THAP11,THRB,THSD7B,THUMPD3,TIAM2,TIE1,TINAG,TJP1,TLR3,TM9SF4,TMC1,TMC5,TMCO1,TMEM126A,TMEM135,TMEM215,TMEM33,TMEM42,TMEM47,TMEM67,TMIE,TMPRSS11E,TMPRSS2,TMPRSS3,TMPRSS5,TMX2,TNN,TNNT2,TOX2,TP53I11,TPD52,TPP2,TPT1,TRAK1,TRIM13,TRIM45,TRIP11,TRRAP,TSGA10IP,TSPAN18,TSPYL5,TSSK2,TTLL10,TTLL4,TTLL9,TULP3,TULP4,TYRO3,TYROBP,UAP1L1,UBE3A,UCK2,UCKL1,UNC79,USO1,USP2,USP21,USP25,USP36,VAV2,VCAN,VDAC3,VEZF1,VIPR1,VNN1,VPS8,WDR49,WDR7,WDR73,WEE1,WIPF1,WIPI2,WNT7A,WWC2,XKR7,XPR1,YWHAG,ZBTB1,ZBTB22,ZBTB25,ZCCHC5,ZFAND2B,ZFAND3,ZFHX3,ZFHX4,ZFP36L2,ZHX1,ZMYND19,ZNF12,ZNF148,ZNF318,ZNF354A,ZNF384,ZNF827
2	Morphology of cells	4.55E-09	219	ABCG8,ACTR2,ADAMTS13,ADD1,AGA,ANKRD2,AQP4,ARHGEF12,ARHGEF2,ARNTL,ARX,ATE1,ATF2,ATP2B2,ATP6AP1,ATP6V0D2,B4GALT1,BFSP1,BLZF1,BRCA2,BTRC,BUB3,C1GALT1,C1QA,CABP4,CAP1,CASP3,CASQ2,CCDC169,SOHLH2,CCKAR,Ccl7,CD40,CD47,CD81,CDC14A,CDC25B,CDC42EP5,CDH23,CDKN2B,CFLAR,CHST15,CIT,CLCA1,CLNS1A,COL12A1,COL2A1,COL3A1,COMMD9,CREBBP,CSNK2A1,CTGF,CTNNB1,DCC,DCK,DEK,DERL2,DKK3,DMPK,DMRT1,DOCK3,DPYSL4,DSE,Danoust,DYSF,EFNA5,EIF4E,EIF4EBP2,ELK1,EMD,EPHB1,ERBB2,ESPL1,ESR1,ETV5,FAIM2,FAT1,FEN1,FGFR2,FIS1,Foxe3,FOXO3,FOXO4,GAN,GATA5,GCNT1,GLTSCR2,GNAS,GP1BA,GPR126,GPR34,GPR55,GPRIN2,GRIN2A,GRK1,GRM1,GSX2,GUCY2F,HAND1,HES5,HIP1,HIVEP2,HNF4A,HRH2,HTR2B,ICAM1,IGFBP2,IL6R,IL6ST,IL7,INPP5B,INSR,IREB2,ITPA,ITPKC,KCNA5,KIAA1522,KIAA1549,KIRREL,LCAT,LCP1,LIG1,LILRB4,LPAR1,LPAR4,LRP8,LYN,MADD,MAG,MAN2A2,MAP3K1,MAPK1,mir-10,mir-130,mir-30,MLANA,MLLT4,Mt1,MYBL2,NAGLU,NBN,NDUFV2,NKX6-1,NOLC1,NTRK3,NXF2,NXF2B,OLIG2,OPN4,PAK1,PEX11A,PICK1,PIP5K1A,PIWIL1,PLA2G15,PLEC,PLK1,Pln,PLSCR1,PMP22,POU2AF1,PPARG,PRKCDBP,PRLH,PROC,PSME3,PTPN22,PYCARD,RAD18,RB1,RCAN2,RECK,RGS2,RPS6KA2,RTN4,RYR2,SEC23IP,SEPT11,SGCG,SIRT7,SKIL,SLC12A5,SLC17A8,SLC6A6,SLC7A1,SMARCA4,SNCA,SNTB2,SPAG16,SPIB,STMN3,SYK,SYNE2,SYTL4,TAF10,TBXA2R,TEKT4,THRB,TIE1,TJP1,TLR3,TNNT2,TPD52,Tpm1,Tpm3,TPP2,TSSK2,TTYH1,TYRO3,TYROBP,UBE3A,USO1,USP2,VAV2,VAX2,VCAN,VDAC3,VEZF1,WIPF1,WNT7A,YWHAG
3	Organization of cytoplasm	1.34E-06	148	ACTR2,ADD1,AFG3L2,ANXA8,ANXA8L1,AP1G1,ARHGEF2,ATF2,ATL3,B9D2,BAIAP2,BLZF1,BSG,C1QA,CAP1,CCKAR,CD47,CD81,CDC20,CDC25B,CDC42EP1,CDC42EP4,CDC42EP5,CDK5RAP2,Cend1,LOC100911402,CEP72,CIT,CTGF,CTNNB1,DAPK1,DCC,DKK3,Dmd,DOCK2,DPYSL4,Dst,EDNRA,EFNA5,EIF4EBP2,ELK1,EMD,ENC1,EPHB1,EPHB6,ERBB2,ESPL1,ESPN,ESR1,F13A1,FANCG,FAT1,FCER1A,FEZF2,FGFR2,FHL3,FIS1,GAN,GAS6,GNAS,GPR126,GSN,HNF4A,HRH2,ICAM1,IGSF9,IL1R1,INOUSR,KCTD13,KIF2B,KIRREL,LAMA5,LCP1,LIG1,LPAR1,LRP8,LRRC16A,LYN,MAG,MAP3K1,MAPOURQUOI1,MAPK10,MAPK3,MAPKAPK5,MINK1,mir-181,mir-29,MTMR12,MYO1F,MYO6,NAGLU,NEU3,NEU4,NPHP4,NPY,NTRK3,PACSIN2,PAK1,PDE4DIP,PEX11A,PIK3C2A,PIK3C3,PIP5K1A,PLEK,PLK1,PMP22,POU3F2,PPARG,PRKAG3,PRMT1,PROC,PSRC1,RAB43,RAB6A,RALBP1,RB1,RGS2,RHOQ,RND2,RNF5,RNF6,RTN4,SEC23IP,SEPT11,SERPINC1,SKIL,SLITRK1,SNCA,SPAG16,SPHK1,SPTAN1,SS18,STK35,STMN3,SYK,TGM2,TIAM2,TIMM50,TMEM135,TMEM67,TNN,Tpm1,TPPP,TSSK2,TTYH1,UBE3A,VAV2,VAX2,WIPF1,WNT7A
4	Cell death	1.61E-06	339	ACER2,ACSL4,ADAMTS13,ADD1,AFG3L2,AGA,AGTR1,Agtr1b,AICDA,AKAP1,AMPD3,ANXA11,AP1G1,APLN,ARHGEF2,ARID3B,ARNT,ARNTL,ARX,AS3MT,ASAH2,ATF2,ATP2B2,ATP2B4,ATP6AP1,ATP6V0D2,AVP,B4GALT1,BMP1,BNIPL,BRCA2,BRF1,BSG,BTRC,C1QA,CA4,CALCB,CASP3,CASQ2,CBX7,CD226,CD40,CD47,CD80,CD81,CD96,CDC14A,CDC20,CDC25B,CDX1,CFLAR,CIDEA,CIT,CLNS1A,COL2A1,COPS5,CPB2,CREBBP,CSNK2A1,CST3,CTBP2,CTGF,CTNNB1,CYP2J2,DAPOURQUOI1,DAXX,DONCC,DCK,DCUN1D3,Defb1,DEK,DKK3,DLST,DMRT1,DNAJB1,DNMT3A,DNMT3B,DOCK2,DPYSL4,Dst,DYSF,EDNRA,EEF1D,EFNA5,EIF4E,ELK1,EMD,ENC1,EPHB6,ERBB2,ESPL1,ESR1,ETV5,EXOC2,F13A1,FABP1,FAIM2,FANCG,FBXO32,FCER1A,FEN1,FGFR2,FGFR4,FIS1,FKBP5,Foxe3,FOXO3,FOXO4,GAS6,GCNT1,GHRH,GHRHR,GLI3,GLTSCR2,GNAL,GNAS,GNB2L1,GNRHR,GRIA2,GRIN2A,GRK1,GRM1,GSN,GSTA1,HAND1,HDAC9,HIP1,HLTF,HNF4A,HNRNPA1,HPGDS,HRH2,HSPA5,HSPBAP1,HTR2B,ICAM1,IGFBP2,IL18R1,IL1R1,IL21R,IL24,IL6R,IL6ST,IL7,IMMT,ING3,INSR,INTS3,IREB2,IRF7,KCNA5,KCND3,KCNIP3,KCNQ2,KEAP1,KLB,KLK1,LAIR1,LAMA5,LCAT,LGALS7,LGALS7B,LIG1,LPAR1,LRP8,LY86,LYN,MADD,MAFK,MAG,MAP3K1,MAP3K3,MAPK1,MAPK10,MAPK3,MBTPS1,MED21,mir-1,mir-10,mir-130,mir-17,mir-181,mir-204,mir-22,mir-29,mir-296,mir-30,mir-34,mir-7,MLANA,MLH1,MMP1,MMP8,MRE11A,MSH3,MSI2,Mt1,MTF1,MTPN,MYBL2,MYO6,MZF1,NAGLU,NBN,NCL,NCOA4,NDUFV2,NEU3,NEUROG1,NKX2-6,NKX6-2,NNAT,NOLC1,NOTCH4,NOVA1,NPHP4,NPY,NR5A2,NRG2,NTRK3,NUP93,NXF2,NXF2B,OAZ1,Olfr380,P4HB,PACRG,PAK1,PARP16,PCDHGA2,PDCD6,PF4,PFKFB1,Pgap2,PHF1,PICK1,PIK3C3,PIWIL1,PKP3,PLEC,PLEKHF1,PLK1,PLSCR1,PMEPA1,PMP22,POU2AF1,PPARG,PPM1A,PPP1R11,PPP2R1A,PRCC,PRKAB1,PRKCG,PRMT1,PROC,PSME3,PTGS1,PTPN22,PYCARD,RAD18,RAD51,RAD51C,RALBP1,RASSF3,RB1,RCAN2,RECK,REPS2,RNF34,RNF5,RPL37,RPS6KA2,RRM2,RTN4,RUNX1T1,RXFP2,RYR2,SCYL3,Serbp1,SERPINC1,SET,SGCG,SIGMAR1,SIRT7,SKIL,SLAMF6,SLC1A2,SLC20A1,SLC2A1,SLC6A6,SMARCA4,SNCA,SNRK,SOX17,SPAG16,SPAG5,SPHK1,SPHK2,SPIB,SPN,ST18,STOM,STOML2,SYK,TAF10,TANK,TBXA2R,Tcf7,TDGF1,TFDP1,TFDP2,TGM2,THRB,TIMM50,TINAG,TLR3,TMEM214,TNNT2,TP53I11,TPD52,TPP2,TPT1,TRIM13,TRIM28,TYRO3,TYROBP,UBE3A,UNC119,USP2,VAV2,VCAN,VDAC1,VIPR1,WEE1,WIPF1,WNT7A,WTAP,XPR1,YWHAG,ZFP36L2,ZNF148
5	Expression of RNA	5.09E-06	203	ABCF1,ABHD14B,ABLIM1,ACTR2,AFF3,ANK2,ARID1B,ARNT,ARNTL,ASCL2,ASCL3,ATF2,ATP2B4,AVP,BLZF1,BMP3,BRCA2,BRF1,BTRC,CAND1,CBX7,CD40,CD47,CDC25B,CDK5RAP2,CDKN2B,CDX1,CFLAR,COL2A1,COPS5,COPZ1,CREBBP,CRLF3,CTBP2,CTGF,CTIF,CTNNB1,DAPK1,DAXX,DDX54,DEK,DKK3,DNAJB1,DNMT3A,DNMT3B,DNMT3L,EEF1D,EIF3K,EIF4E,EIF4EBP2,ELK1,ENC1,EPHB1,ERBB2,ESR1,ETV5,FGFR2,Foxe3,FOXO3,FOXO4,GAS6,GATA5,GHRH,GLI3,GNB2L1,GNRHR,GTF2A1,HAND1,HDAC9,HEATR1,HES5,HIP1,HIVEP2,HIVEP3,HLTF,HNF4A,HNRNPA1,HTR2B,ID4,IL6R,IL6ST,INOUSR,IREB2,IRF7,IRF9,JAZF1,KCNIP3,KCP,KCTD13,KEAP1,LIMD1,LPAR1,LRP8,LYN,MAFK,MAML3,MAP3K1,MAP3K3,MAPK1,MAPK3,MC4R,MED21,MED23,MEIS2,mir-1,mir-7,MLH1,MRE11A,MTF1,MTIF2,MTPN,MURC,MYBL2,MYO6,MZF1,NCBP2,NCOA4,NEUROG1,NKX2-6,NKX6-1,NKX6-2,NOLC1,NOTCH4,NR5A2,NR6A1,OLIG2,OVOL2,PAK1,PDE2A,PF4,PLK1,PLSCR1,PMF1,BGLAP,POU2AF1,POU3F2,PPARG,PPM1A,PRKAB1,PROC,PSRC1,PTF1A,RALBP1,RB1,RBBP8,RCAN2,RFX5,RGS2,RHOQ,RNF6,RPL17,RPL37,RPS29,RPS6KA2,RUNX1T1,RUVBL1,RXFP2,SARS,Scaf1,SERTAD2,SET,SIM1,SIRT7,SKIL,SLC20A1,SMARCA4,SNAPC5,SOX17,SP2,SPIB,SPZ1,SS18,ST18,SYK,TAF10,TANK,TCEA2,Tcf7,TDGF1,TFDP1,TFDP2,TFG,TGM2,THRB,TLR3,TOX2,TRIM13,TRIM28,TRIP11,TRRAP,UBE3A,USP2,USP21,VAV2,VAX2,VEZF1,WNT7A,ZFHX3,ZFP36L2,ZHX1,ZNF12,ZNF148,ZNF354A,ZNF384

Table S8: Top 10 canonical pathways enriched in differentially methylated genes of the green module

	Functions	p-value	Number of genes	Genes
1	Influx of amino acids	4.24E-5	2	SLC1A2,SLC5A6
2	Differentiation of plasma cells	1.45E-4	3	IL21R,SIGLEC10,SPIB
3	Anxiety	2.38E-4	5	GRIN2A,KCNIP3,NDST3,PAK1,SLITRK1
4	Processing of protein fragments	7.45E-4	3	ADAMTS13,CHST8,GLI3
5	Differentiation of cells	1.01E-3	17	ARNT,ARPP21,DPYSL4,FECH,GLI3,ICAM1,IL21R,NKX2-6,PAK1,PEX11A,SIGLEC10,SLAMF6,SLC1A2,SPIB,SYNE1,TNN,Zfp35
6	Injury of brain	1.17E-3	3	ADAMTS13,GRIN2A,SLC1A2
7	Quantity of dendrites	1.31E-3	2	DPYSL4,PAK1
8	Vascularization of extraembryonic tissue	1.31E-3	2	ARNT,ICAM1
9	Differentiation of lymphocytes	1.31E-3	7	GLI3,ICAM1,IL21R,SIGLEC10,SLAMF6,SPIB,Zfp35
10	Cytotoxicity of lymphocytes	1.31E-3	4	ICAM1,IL21R,PAK1,SLAMF6

Table S9: List of primers used for QPCR

**Brain/T cells (QMeDIP)**	
pax6-Forward	5'-TTGTATCCTTGGCTGAGGGG-3'
pax6-Reverse	5'-GTTGGAACCCCAACTCACAC-3'
clip3-Forward	5'-CTAGGATGCTCACCCTCTGG-3'
clip3-Reverse	5'-TCCCCTGAAGCAGATGACTC-3'
pak1-Forward	5'-CAGTGGTGGGAGAAGAGGAG-3'
pak-Reverse	5'-AGAGTAAGGGCGGGGAAAAA-3'
**Brain (QMeDIP)**	
kcnab3-Forward	5'-AGTCTATCTTGGCGGAGTCG-3'
kcnab3-Reverse	5'-GTTTCTGGTACCTCCCTCCC-3'
kcnc3-Forward	5'-GTGCAATCCCACCTCCTCTA-3'
kcnc3-Reverse	5'-ACACGAGCTGATTGGACTGA-3'
l1cam-Forward	5'-TTTGTGGACATGCCTCTTGC-3'
l1cam-Reverse	5'-ACAAGGGTGGTGAGGAACAT-3'
id4-Forward	5'-GCCCGAAGCAAAAGACAAGA-3'
id4-Reverse	5'-GGACAGCGATCCACCTTAGT-3'
at2b3-Forward	5'-AAGCTAGTGATGGGCACTGA-3'
at2b3-Reverse	5'-GAGGGTTTGTTAGGTGGGGA-3'
rpl22l1-Forward	5'-AAGCTAGTGATGGGCACTGA-3'
rpl22l1-Reverse	5'-GAGGGTTTGTTAGGTGGGGA-3'
dnmt1-Forward	5'-CTCTGATAGCTCCAGGATCGC-3'
dnmt1-Reverse	5'-GTTCGAGTCCCGCTGTCAA-3'
nfkb1-Forward	5'-CTGTAACTCCGGAGGGAACC-3'
nfkb1-Reverse	5'-CACACGATACCCTCCTTCCG-3'
Avpr1b-Forward	5'-GTGTCACCTCTCAGAAGCCC-3'
Avpr1b-Reverse	5'-AGAGGCTCAGGGACCAAAGA-3'
Ank3-Forward	5'-AACTTGCATTGGCATCCGAC-3'
Ank3-Reverse	5'-GTACCATTGGCTGGGTACCTG-3'

oprm1-Forward	5'-GGCAGTTGAGTCGGAAGAGT-3'
oprm1-Reverse	5'-ACATTGCAGAGAAGGGTGGG-3'
ntrk2-Forward	5'-CTCCAGACAAAAGCAAGGCG-3'
ntrk2-Reverse	5'-TGAACTCCCACATGCTGCTG-3'
htr1a-Forward	5'-GACCCAGAAGAAGGAGGCAC-3'
htr1a-Reverse	5'-GCTAGTCTCCCTTCCTCCCA-3'
Grin1-Forward	5'-GCTGGAGTGTGTGTACTGGA-3'
Grin1-Reverse	5'-CCCCCAGAGGTATAGAGGGA-3'
**T cells (QMeDIP)**	
Ank2-Forward	5'-AGGTGTTCACATGTGTGGGG-3'
Ank2-Reverse	5'-AGGGACAGACACCACCACTA-3'
Dip2C-Forward	5'-GCAACACCATTATAGACAGGCATT-3'
Dip2C-Reverse	5'-GCTTCCTTTGGTCTTGGTCATTTC-3'
epb41l4b-Forward	5'-GTGAAAGGGAACGTGTGGGA-3'
epb41l4b-Reverse	5'-GGCCTGTAGCCCATTCTTGT-3'
piwil1-Forward	5'-GCAAGCCATTCCACCCCTTA-3'
piwil1-Reverse	5'-CATCTCAGCTAGCACGGACC-3'
rp6kc1-Forward	5'-ACTTTTCCTTCCCACCCTGC-3'
rp6kc1-Reverse	5'-CGAACTTGGAGACAGTGGTGA-3'
serpinb12-Forward	5'-CTGGTTCCTTTAGCAGCCGA-3'
serpinb12-Reverse	5'-CTGGTTCCTTTAGCAGCCGA-3'
snora41-Forward	5'-CCCACAAGCAGTAAGAAACCATC-3'
snora41-Reverse	5'-GGTGCATACTGATCTGACCCC-3'
tkt-Forward	5'-TCCTTGACCTGTTGGTGAGC-3'
tkt-Reverse	5'-ACCTGGGACTGGAGTGATGT-3'
vomr28-Forward	5'-ACTCAGTTGGGCTAAGTAACCTG-3'
vomr28-Reverse	5'-CTCCTCTTGGGATGATTTGGCT-3'
**Brain (mRNA)**	
KCNAB3-Forward	5'-GCCCTATACTGGGGGACATC-3'

KCNAB3-Reverse	5'-GCTTGTTCACACACAGGAGG-3'
KCNC3-Forward	5'-CGCTTTTTGAGGACCCCTAC-3'
KCNC3-Reverse	5'-ACCGTCTTGTTGCTGATGTG-3'
ID4-Forward	5'-TGCAGTGCGATATGAACGAC-3'
ID4-Reverse	5'-TGCTGACTTTCTTGTTGGGC-3'
NFKB1-Forward	5'-ACGACGATCCTTTCGGAACT-3'
NFKB1-Reverse	5'-TCCTCTCTGTTTCGGTTGCT-3'
IKBKB-Forward	5'-TGAGTGACATAGCATCGGCT-3'
IKBKB-Reverse	5'-CCAGCTCCTTGGCATATCCT-3'
IL21R-Forward	5'-CAAGAGTGTGGCAGCTTTGT-3'
IL21R-Reverse	5'-CGTCCTGAGAAGGTCACAGT-3'
dnmt3b-Forward	5'-AATCTGCACAGAGCCAGTCT-3'
dnmt3b-Reverse	5'-AGAGCCATCTCCATCATCCG-3'
dnmt3a-Forward	5'-GTGTGTCGAGAAGCTCATGC-3'
dnmt3a-Reverse	5'-TCGTAGATGGCTTTGCGGTA-3'
dnmt1-Forward	5'-GTCGGACAGTGAGACCATGA-3'
dnmt1-Reverse	5'-AAGTGAGACGTGATGGTGGT-3'
tubb5-Forward	5’-TAGAACCTTCCTGCGGTCGT-3’
tubb5-Reverse	5’-TTTTCTTCTGGGCTGGTCTC-3’
